# Benchmarking Cost-Effective DNA Extraction Kits for Diverse Metagenomic Samples

**DOI:** 10.3390/ijms262311616

**Published:** 2025-11-30

**Authors:** Andrey Sobolev, Daria Sibiryakina, Elizaveta Chevokina, Darya Slonova, Daria Yurikova, Svetlana Kozlova, Anna Trofimova, Vasiliy Zubarev, Alexander Kiselev, Olga Konovalova, Dmitry Sutormin, Artem Isaev

**Affiliations:** 1 The Center for Bio- and Medical Technologies, Moscow 121205, Russia; chorzoww@gmail.com (A.S.); d.sibiryakina@yandex.ru (D.S.); lizcross13@gmail.com (E.C.); sveta.cozlowa2010@yandex.ru (S.K.); annat@genebiology.ru (A.T.); vmz2home@gmail.com (V.Z.); 2 Gamaleya National Research Centre of Epidemiology and Microbiology, Moscow 123098, Russia; 3 Marine Research Center of Lomonosov Moscow State University, Moscow 119607, Russia; d.urikova@marine-rc.ru (D.Y.); o.konovalova@marine-rc.ru (O.K.); 4 Shirshov Institute of Oceanology, Russian Academy of Sciences, Moscow 117997, Russia; ad-kiselev@mail.ru; 5 Faculty of Biology, Lomonosov Moscow State University, Moscow 119234, Russia

**Keywords:** DNA purification, benchmark, 16S sequencing, metagenomics, cost-effectiveness, environmental samples

## Abstract

Extraction of high-quality microbial DNA remains a critical bottleneck in metagenomic research. Environmental samples often produce fragmented DNA and are prone to contaminations that interfere with downstream sequencing, while widely used commercial kits can be prohibitively expensive. Therefore, systematic evaluation of cost-effective alternatives is essential to support large-scale metagenomic studies. In this work, we benchmarked eight commercial DNA extraction kits from Magen, SkyGen, and Sileks against Qiagen reference kits. Four representative sample types were analyzed: freshwater, seafloor sediments, Pacific oyster (*Magallana gigas*) gut microbiome, and mammalian feces. DNA yield, integrity, purity, PCR inhibitor content, and eukaryotic DNA admixture were assessed. Microbial community composition, alpha diversity, reproducibility, and contamination (“kitome” and “splashome”) were further evaluated using 16S rRNA amplicon sequencing. We revealed that several alternative kits performed comparably or better than the Qiagen reference standard. Magen Soil and Magen Bacterial provided high yields and reproducibility, though the latter produced more fragmented DNA. SkyGen Stool excelled with host-associated samples, while Sileks Soil and Metagenomic kits preserved higher diversity in sediments. Magen Microbiome consistently underperformed. This study identifies multiple cost-effective DNA extraction strategies and provides practical guidance for selecting balanced DNA purification methods for different sample types.

## 1. Introduction

The Earth’s biosphere harbors an immense diversity of prokaryotes, estimated at 10^30^ cells and 10^12^ species [1], yet only a tiny fraction have been identified and studied. Our knowledge remains particularly limited in hard-to-access habitats such as deep-sea sediments and the Arctic regions [2,3,4,5,6]. Nevertheless, prokaryotic communities play a fundamental role in maintaining marine and terrestrial ecosystems [7,8]. To understand complex ecological interaction networks, it is necessary to determine both the community composition and the functional roles of individual microorganisms [8,9]. Although a diverse range of biochemical adaptations has been characterized in known microbes, the discovery of novel species could provide even deeper insights for both basic and applied research [10,11]. However, most microbial species belong to the so-called “microbial dark matter” and are unculturable under laboratory conditions [12].

Metagenomics enables qualitative and quantitative analysis of microbial communities through direct sequencing of genetic material extracted from environmental samples, making it an invaluable tool for exploring previously inaccessible microbial diversity [13,14]. Metagenomic sequencing methods and data analysis algorithms are constantly being improved. Among these, amplicon sequencing of the hypervariable regions of the 16S ribosomal RNA (rRNA) gene is the most widespread approach [15]. It provides a cost-effective, high-throughput method for taxonomic classification of sequenced DNA fragments and enables assessment of both alpha- and beta-diversity, representing microbial diversity within a single sample (alpha-diversity) or between samples (beta-diversity) [15,16,17]. Over the past few decades, 16S rRNA sequencing has become the gold standard for taxonomic identification and underpins modern microbial classification systems [18]. However, this method cannot provide functional insights. To partially address this gap, computational tools such as PICRUSt and Tax4Fun have been developed to predict functional traits based on 16S rRNA data [19,20,21]. Yet, their predictive accuracy remains limited, particularly for poorly characterized environments beyond the human gut microbiome.

A more powerful alternative is shotgun metagenomic sequencing, which allows reconstruction of genomic fragments (contigs) or near-complete microbial genomes and enables identification of functional gene clusters [22,23,24,25]. This approach achieves optimal results when short-read sequencing (e.g., Illumina, BGI) is combined with long-read technologies (e.g., Oxford Nanopore, PacBio) in a hybrid assembly strategy, yielding more contiguous and accurate genome assemblies [26,27,28,29].

Short-read and especially long-read metagenomic sequencing requires high-quality metagenomic DNA, which is challenging to extract, since environmental samples often produce DNA of low yield and purity. DNA fragmentation is another problem significantly affecting the quality of shotgun assemblies. Moreover, sample processing and DNA extraction protocols can substantially alter the observed microbial community composition [30,31,32,33,34]. Multiple benchmarking studies were conducted to evaluate these biases and identify optimal methods for reproducible DNA extraction and sequencing across diverse sample types [30,32,34,35,36,37,38,39,40,41]. Nevertheless, such systematic evaluations remain particularly scarce for marine environments, sediments, and host-associated microbiomes.

To address this methodological gap, we recently conducted a comprehensive benchmarking study evaluating the most widely used DNA extraction protocols and commercial kits for marine metagenomics. Our results demonstrated that the PowerSoil and PowerFecal kits (Qiagen) consistently yield high-quality DNA suitable for metagenomic sequencing across diverse marine sample types [42]. However, these kits are among the most expensive commercially available options. This cost factor could present a significant practical constraint, particularly for small research laboratories with limited budgets or large-scale projects requiring high-throughput processing.

To aid in the selection of cost-effective environmental DNA purification methods, we expanded our previous benchmarking procedure by evaluating the performance of eight additional DNA extraction kits against the established “gold-standard” references [42]. Our analysis incorporated four ecologically representative sample types: filtered freshwater, seafloor sediments, the microbiome of the model invertebrate Pacific oyster (*Magallana gigas*), and mammalian fecal samples collected from a dog (*Canis lupus*). We have implemented a comprehensive DNA quality assessment protocol, evaluating DNA yield, fragment size distribution, PCR inhibitor content, and eukaryotic DNA admixture. In addition, we performed 16S amplicon sequencing for each sample and evaluated the resulting microbial community composition, technical reproducibility, and kit-associated contaminants (“kitome”). Notably, our results identified several alternative extraction kits that demonstrated equivalent or superior performance to the “gold-standard” reference methods. These kits can be used for both 16S amplicon and shotgun or long-read metagenomic sequencing.

## 2. Results

### 2.1. Design of the Study and Samples Processing

**Figure 1:**
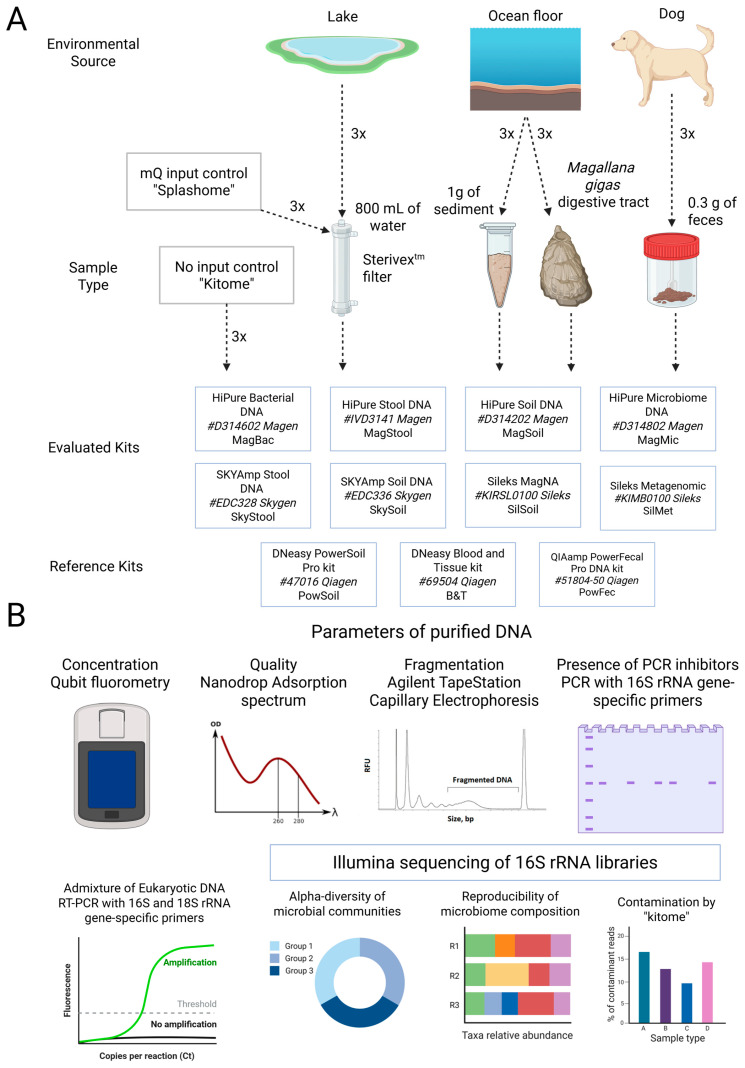
A schematic representation of the methodology of the study. (**A**) Four types of samples were collected. Each sample was processed in three technical replicates with eight commercial DNA purification kits and one reference kit. In addition, two types of negative controls were processed: a no-input control representing the “kitome”, and an mQ water input control representing the “splashome”. (**B**) All samples were evaluated for an indicated set of parameters to select the best DNA purification strategy. This figure is a modified version of a figure previously prepared by the authors and published in [42].

The overall design of the study is depicted in Figure 1 and described in detail in the “Materials and Methods” section. To compare different DNA isolation kits, we processed four representative types of samples: gut microbiota of a marine invertebrate (referred to as “microbiota”), freshwater, seafloor sediment, and mammalian fecal samples. Microbiota was extracted from six individuals of the giant Pacific oyster (*Magallana gigas*) species, collected from Melkovodnaya Bay near Vladivostok, Russia (see Materials and Methods, Figure S1). Their digestive tracts were homogenized and pooled for further processing to minimize sample heterogeneity caused by the biological variability of mollusk individuals. DNA extraction was performed from 300 mg aliquots of the homogenized sample. Freshwater was collected from a pond in Moscow, Russia (see Materials and Methods, Figure S1), and 800 mL aliquots from a single batch were filtered through Sterivex filters using a peristaltic pump. All filters were frozen at −20 °C and processed together within one day. A STET lysis solution containing lysozyme was added to the fragmented filter membranes, and the resulting eluate was used as input for further processing. The sea sediment sample was collected at the littoral zone of the Barents Sea, Russia (see Materials and Methods, Figure S1). The sample was thoroughly mixed to ensure uniformity, and an equivalent of 1 g of dry weight was taken for processing with each kit. Animal fecal samples were collected in Moscow, Russia, from a dog (*Canis lupus*) (see Materials and Methods, Figure S1). The sample was thoroughly mixed to ensure uniformity, and aliquots equivalent to 300 mg of dry weight were taken for DNA extraction.

Each sample was processed in triplicate with eight commercially available DNA extraction kits and a reference kit (PowerSoil for seafloor sediment, microbiota, and fecal material; B&T for freshwater; and PowerFecal for fecal samples). Additionally, two types of negative controls were processed to account for contamination. The “kitome” control represents a sample processed without any input, only with the use of the solutions provided by the kit. This provides information about the microbiota or residual DNA present in the kit. The second negative control, a combination of the “kitome” and “splashome”, uses mQ water, treated identically to freshwater samples, as an input. In addition to the contaminants associated with the kit components, this control provides information about the contamination of laboratory-grade mQ water and Sterivex filters.

The evaluated kits use a variety of methods for cell lysis (enzymatic or chemical lysis, mechanical homogenization), depletion of host DNA, contaminant removal, and DNA purification (silica-based membranes or magnetic beads) approaches (Table 1). Only the B&T reference kit does not require the use of bead beating, and most of the kits provide buffers for the chemical lysis of bacterial cells in addition to mechanical homogenization. Nine kits rely on silica spin columns for DNA isolation and purification, while Sileks Soil and Sileks Metagenomic kits include magnetic beads for this purpose. Sample processing was carried out according to the manufacturer’s instructions with minor modifications. Purified DNA was eluted in a 60 µL volume of elution buffer supplied with the kit and was stored at −80 °C. The samples were analyzed for various DNA characteristics and were used for 16S rRNA sequencing.

To estimate the economic and time expenses associated with each protocol, we also evaluated kit prices (recorded on 10 May 2024 from the official supplier websites) and the overall simplicity of the procedure (accounting for the estimated duration of the protocol). Based on our user experience, reference kits from Qiagen required less processing time. However, when considering cost per sample, these kits also tended to be two to three times more expensive compared to selected alternatives (Table 1). Thus, we set the goal of finding a balanced DNA purification method that accounts for both cost and DNA quality.

### 2.2. DNA Yield

The concentration of the DNA extracted by each kit was measured using the Qubit dsDNA High Sensitivity Assay or Broad Range Assay on the Qubit 4.0 fluorometer. For each type of sample, there were significant differences in the effectiveness of DNA extraction using different extraction kits.

**Figure 2:**
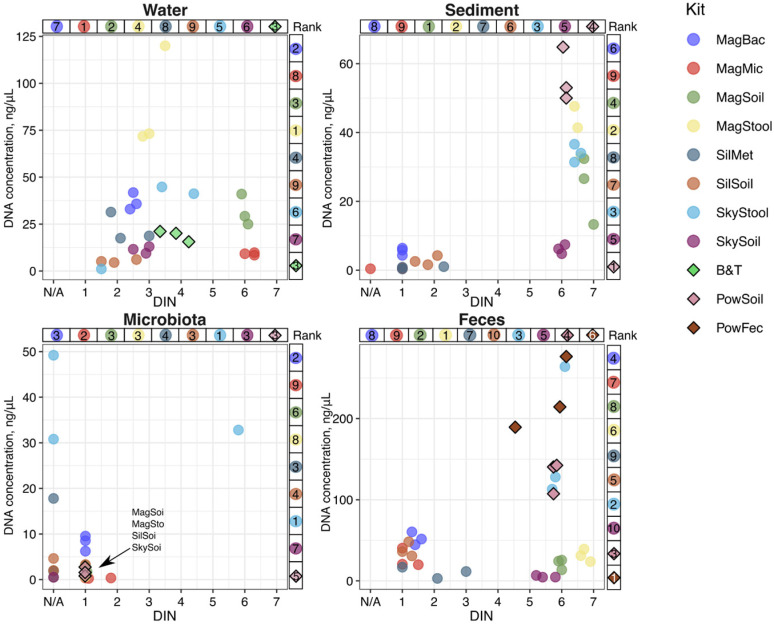
Concentrations and DNA integrity number (DIN) of DNA extracted from different sample types using tested kits. Data for three technical replicates are shown. N/A on the *x*-axis represents samples where DIN could not be measured on TapeStation due to low DNA quality. Samples produced by reference kits are shown with diamond-shaped points with black borders. Kit ranks are shown on the right (for DNA concentration) and on top (for DIN) of each subplot. Lower ranks indicate higher DNA quantity or quality.

For freshwater samples, the Magen Stool extraction kit produced the highest yield of DNA (~75 ng/μL). The Magen Soil, Magen Bacterial, and SkyGen Stool kits yielded approximately the same amount of DNA (~25–50 ng/μL), and these four kits outperformed the commonly used reference B&T kit (Qiagen). The Magen Microbiome and Sileks Soil kits had the lowest yields, with less than 10 ng/μL DNA extracted (Figure 2, Table S1).

For sediment samples, the Magen Stool, Magen Soil, and SkyGen Stool kits demonstrated a fairly high DNA yield of above 30 ng/μL, although they were less efficient than the Power Soil reference kit. Similarly to the results for freshwater samples, the Magen Microbiome and Sileks Soil kits yielded the lowest amount of DNA (Figure 2, Table S1).

For microbiota samples, all kits except the Magen Bacterial and SkyGen Stool kits yielded extremely low quantities of isolated DNA. Surprisingly, the reference Power Soil kit also demonstrated poor results with this sample type (Figure 2, Table S1). Since the amount of DNA extracted using the SkyGen Stool kit exceeded that obtained using other kits, we tested these samples for possible RNA contamination by treating the extracted material with DNase and RNase. The results confirmed the absence of detectable RNA contamination (Figure S2).

Lastly, for mammalian fecal samples, multiple kits produced sufficient DNA yield. The SkyGen Stool Kit demonstrated the highest DNA yield of above 100 ng/μL, which was comparable to the reference Power Soil and Power Fecal Kits. The SkyGen Soil and Sileks Metagenomic kits yielded the lowest amount of DNA (Figure 2, Table S1).

The Magen Bacterial and SkyGen Stool kits showed consistent performance across various sample types. These kits even outperformed the reference kit for freshwater samples. The Magen Stool and Magen Soil kits achieved high concentrations of purified DNA for freshwater and sediment samples, although they were less effective with *M. gigas* and dog fecal samples. The Magen Microbiome, SkyGen Soil, Sileks Metagenomic, and Sileks Soil kits demonstrated low DNA yields with all tested sample types.

### 2.3. DNA Fragmentation

The level of extracted DNA fragmentation was estimated using capillary electrophoresis on a TapeStation 4150 system (Agilent). The results are provided as a DNA integrity number (DIN). A DIN value of 6–7 is generally considered acceptable for long-read sequencing applications, such as those offered by ONT or PacBio technologies. Higher DIN values result in longer sequencing reads and increased quality of data [43,44].

High-quality DNA (DIN > 5.5) was extracted from the freshwater, marine sediment, and dog feces samples using the Magen Soil extraction kit (Figure 2 and Figure S3, Table S1), surpassing the performance of the reference kits. Similarly, SkyGen Stool outperformed the reference kits with the marine sediment, *M. gigas* gut microbiota, and dog feces samples, and showed a DIN > 5.5. Additionally, the SkyGen Soil and Magen Stool kits extracted high-quality DNA (DIN > 5.5) from the marine sediment and dog feces samples, and the Magen Microbiome kit performed well with the freshwater samples (DIN > 6). All samples of *M. gigas* gut microbiota, except one outlier replica obtained with SkyGen Stool, contained low-quality DNA (DIN < 2), which indicates significant DNA degradation and suggests possible contamination with host-derived nucleases.

### 2.4. DNA Purity and Presence of PCR Inhibitors

The purity of DNA samples was assessed using the NanoDrop spectrophotometer. A 260/280 absorbance ratio between 1.8 and 1.9 typically indicates the absence of protein contamination, whereas a 260/230 absorbance in the range of 1.8–2.5 indicates the absence of organic impurities [45].

DNA purity varied for different sample types and different kits used, while also demonstrating technical variability. The 260/230 ratios were generally lower for gut microbiota samples, indicating increased contamination with organic compounds (Figure S4, Table S1). Nevertheless, kits with reliable performance comparable to the reference kits’ performance were available for all sample types. For freshwater samples, the best results were obtained with Magen Stool, Magen Soil, Sileks Metagenomic, and SkyGen Stool kits. For marine sediment samples, Magen Stool, Magen Soil, SkyGen Soil, and SkyGen Stool performed well. The Magen Bacterial kit extracted relatively pure DNA from the microbiota samples; however, it showed lower or average performance with the other sample types. Additionally, Sileks Soil and SkyGen Stool also performed well with the microbiota samples. DNA obtained with Magen Microbiome generally exhibited increased levels of protein and organic contamination, except for the fecal samples, for which the results even outperformed the reference Power Fecal and Power Soil kits. Magen Stool and Sileks Soil were among the top-performing kits for this sample type.

**Figure 3:**
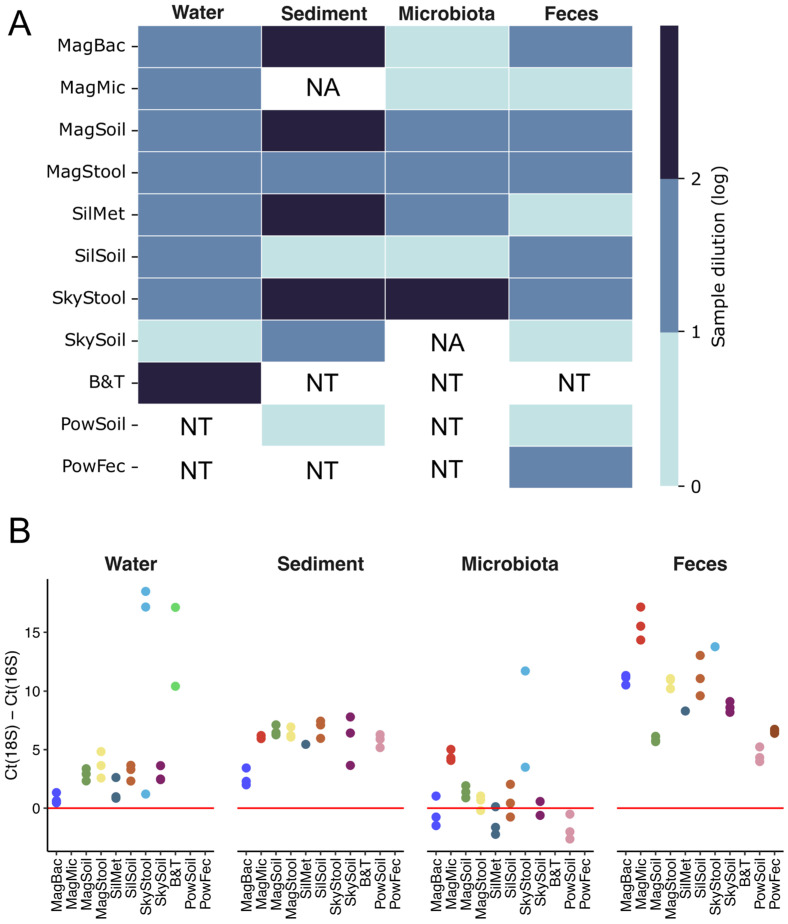
Detection of PCR inhibitors and an admixture of eukaryotic DNA in samples extracted from four sample types using the different kits tested. (**A**)—The presence of DNA inhibitors as estimated by the dilution factor required to achieve detectable amplification of the V3-V4 16S rRNA gene region. A mean of three technical replicates is shown. NA—PCR failed with any dilution; NT—was not tested. Gel electrophoresis results for experimental samples as well as negative controls are provided in Figure S5. (**B**)—Admixture of eukaryotic DNA estimated as a difference between Ct values obtained for 18S and 16S gene primers. Higher positive values indicate a larger predominance of bacterial DNA relative to eukaryotic DNA, while the negative values indicate predominance of eukaryotic DNA. Data for three technical qPCR replicates are shown.

The presence of organic impurities could significantly affect the efficiency of PCR and other enzymatic reactions, which could result in sequencing library preparation failure [46,47]. To assess the presence of PCR inhibitors in the extracted samples, we performed 25 cycles of PCR using degenerate primers for the V3-V4 region of the 16S rRNA gene and Phusion DNA Polymerase (NEB). We assessed PCR efficiency using the original samples as well as 10- and 100-fold dilutions that should have lowered the concentration of inhibitors (Figure 3A and Figure S5). With DNA extracted from the freshwater samples, only the SkyGen Soil kit showed a low level of PCR inhibition, allowing amplification without dilution. For all other experimental kits tested, a 10-fold dilution was sufficient to achieve stable amplification. DNA extracted from marine sediment samples was found to be heavily contaminated with PCR inhibitors: samples extracted with four tested kits (Magen Soil, Magen Bacterial, Sileks Metagenomic, and SkyGen Stool) required a 100-fold dilution to be amplified; Magen Microbiome showed no amplification; and only samples extracted with Sileks Soil and the reference PowerSoil kits generated amplicons without dilution, indicating an efficient removal of inhibitors. DNA extracted from *M. gigas* gut microbiota with Magen Microbiome, Magen Bacterial, and Sileks Soil could be considered inhibitor-free and was amplified with no dilution. DNA extracted with three kits (Magen Stool, Magen Soil, and Sileks Metagenomic) required a 10-fold dilution, and SkyGen Stool required a 100-fold dilution, while PCR completely failed with the DNA extracted with the SkyGen Soil kit. Finally, DNA extracted from a fecal sample using three tested kits (Magen Microbiome, Sileks Metagenomic, SkyGen Soil) as well as a reference PowerSoil kit, was pure enough to be successfully amplified without dilution, and DNA samples produced with other kits required a 10-fold dilution (Figure 3A).

### 2.5. Admixture of Eukaryotic DNA

DNA extracted from certain sample types, particularly gut microbiota, often comprises a mixture of microbial and eukaryotic genetic material. Consequently, while high DNA yield and purity are necessary prerequisites, they do not guarantee successful metagenomic sequencing outcomes, as host-derived DNA can dominate the sequencing library and obscure microbial signals [42,48,49]. In order to assess the presence of eukaryotic DNA in samples, we used quantitative PCR (qPCR) with gene-specific primers for 16S and 18S rRNA genes. The relative signal from 18S and 16S amplicons could represent a valuable metric to estimate the ratio of microbial-to-eukaryotic DNA in a sample. We subtracted a threshold cycle value for 16S amplicon (Ct(16S)) from a threshold cycle value for 18S amplicon—Ct(18S). Positive values indicate a higher abundance of 16S rRNA genes, and a 3-cycle difference is roughly equivalent to a 10-fold difference in gene copy number. Due to the presence of PCR inhibitors, we used 10-fold diluted samples to produce a qPCR signal.

All water samples produced Ct(18S)-Ct(16S) values above 0, indicating a prevalence of bacterial DNA. The highest values were obtained with SkyGen Stool and the reference Blood&Tissue kits, while the Magen Bacterial kit demonstrated the lowest results (Figure 3B, Table S1). Similarly, marine sediment samples were dominated by bacterial DNA (~100x more 16S gene copies), with comparable values obtained for all kits, including the reference, PowerSoil. Again, the Magen Bacterial kit was an outlier, with the lowest Ct(18S)-Ct(16S) value. In contrast, the *M. gigas* gut microbiota samples had low Ct(18S)-Ct(16S) values, with many samples containing more 18S than 16S gene copies (Magen Bacterial, Sileks Metagenome, and the reference PowerSoil kit). The best performance was achieved by the Magen Microbiome and SkyGen Stool kits, indicating higher selectivity towards the extraction of microbial DNA. Fecal samples were dominated by microbial DNA (~100–10,000x excess of 16S gene copies), with the Magen Microbiome and SkyGen Stool kits demonstrating the highest results. Surprisingly, the reference PowerSoil and PowerFecal kits showed the lowest Ct(18S)-Ct(16S) values; however, DNA extracted with these kits can still be considered suitable for microbial metagenomics (Figure 3B, Table S1).

Overall, multiple kits performed comparably well or even better than the reference kits. In particular, the Magen Microbiome and SkyGen Stool kits demonstrated the highest selectivity towards microbial DNA. In contrast, the Magen Bacterial and Sileks Metagenome kits were associated with decreased microbial DNA selectivity for three out of four sample types.

### 2.6. Contamination Levels and Determination of “Kitomes” and “Splashomes”

Contamination with non-sample-specific DNA could represent a significant challenge for microbial metagenomics. One unavoidable source of contamination is DNA or bacterial cells present in the kit reagents, collectively named as the “kitome”. DNA accidentally introduced from a laboratory environment (water, air, pipettes, research personnel, or cross-sample contamination could be named the “splashome” [42,50]. Contamination with external DNA is particularly deleterious for low-biomass samples and may result in distortion of the microbiome composition or false-positive detection of microbial taxa [50,51].

To evaluate the level of contamination introduced by different tested DNA extraction kits, we isolated DNA from two types of negative controls: a no-input control for “kitome” determination, and a filtrate of sterile laboratory water, treated according to the same protocol as other water samples, for estimation of the “kitome” and “splashome” in combination. Next, we characterized the microbiome composition of the negative controls as well as the experimental samples by sequencing the V3–V4 16S rRNA gene region amplicons using NovaSeq 6000 and processed the sequencing data to the level of operational taxonomic units (OTUs). Most samples were sequenced to saturation (Figure S6).

**Figure 4:**
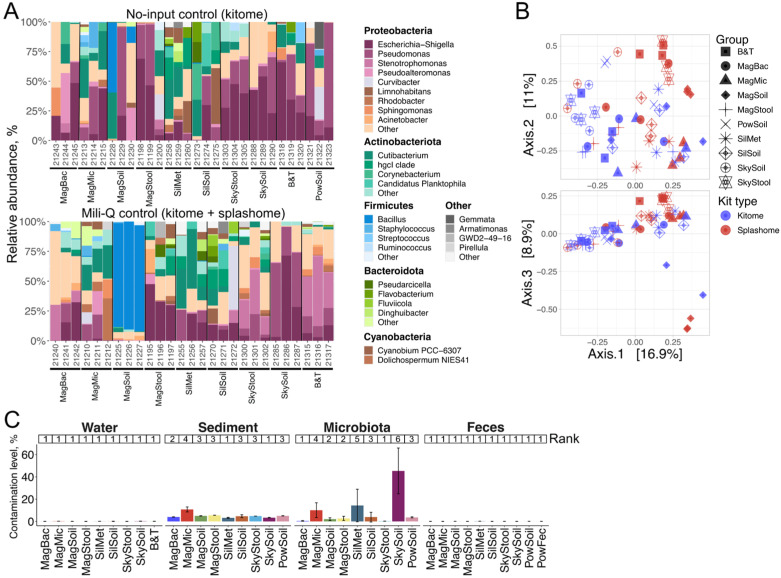
Analysis of “kitomes” and “splashomes” with 16S metagenomics and contamination levels of samples extracted by different kits tested. (**A**)—Relative abundance of microbial orders in “splashomes” (laboratory water group) and “kitomes” (laboratory control and feces groups) prepared with tested DNA extraction kits. Data for three technical replicates are shown. Bacterial genera with relative abundances > 1% are shown. Numbers indicate internal labels for different replicates (BioProject ID PRJNA1183575). (**B**)—PCoA of Bray–Curtis dissimilarity of negative controls processed with different DNA extraction kits, projections of principal components 1 and 2 (above) and 1 and 3 (below) are shown. (**C**)—Relative levels of contamination for different groups of samples processed with tested kits. Bars and error bars represent mean relative contamination and standard deviation for three biological replicates, respectively. Numbers above each bar represent kit ranking. Lower rank indicates lower sample contamination.

Control samples acquired significantly lower numbers of 16S reads than test samples (Figure S7), which indicates limited contamination. Samples processed with Magen Microbiome, Blood & Tissue, and Sileks Metagenome had decreased ratios of control-to-sample median read numbers (<3.1 vs. >4.5 for other kits), potentially indicating relatively increased contamination (Table S2). The composition of the negative controls was highly variable and kit-specific (Figure 4A). The most abundant genera were Escherichia, Pseudomonas, and Stenotrophomonas (all genera belong to Gammaproteobacteria), Cutibacterium (Actinomycetota), and Flavobacterium (Bacteroidota) with a total relative abundance of more than 50% in many controls. Additionally, Bacillus (Bacillota) was abundant in the controls processed with the Magen Soil kit (Figure 4A). All these genera were highlighted as potential contaminants in previous studies [42,51,52].

By comparing the “kitome” and “splashome” controls, it is possible to estimate the effect of the initial water sample processing (filtering, manipulation with Sterivex filters) on contamination. Analysis of the beta-diversity of the negative controls revealed that kits can be classified into two general groups: those with a similar composition of contaminants for both types of negative controls and those with a significantly different composition (Figure 4B). For Magen Soil, SkyGen Stool, and the reference Blood&Tissue kits, contamination introduced with milli-Q water or through filter processing was greater or comparable with kit-derived contamination. For all other kits, internal contamination was presumably stronger, as it was not affected by the “splashome” in the control preprocessing.

To determine the impact of contamination with external DNA on the composition of natural samples, we identified contaminant bacterial OTUs using the Decontam algorithm [53]. Contamination levels, calculated as a total relative abundance of contaminant OTUs, varied significantly across samples (Figure 4C and Figure S8). Freshwater and fecal samples were less affected by contamination, with average contamination levels of 0.11 ± 0.07% and 0.05 ± 0.07%, respectively. Marine sediment and *M. gigas* gut microbiota samples contained relatively high average levels of contamination (5.17 ± 2.28% and 9.15 ± 15.6%, respectively), reaching 45.27% and 14.27% for microbiota samples obtained with the SkyGen Soil and Sileks Metagenomic kits. At the same time, some DNA extraction kits demonstrated low contamination levels for this type of sample, reaching 0.2% and 0.4% for samples obtained with the SkyGen Stool and Magen Bacterial kits, respectively. Marine sediment and microbiota samples processed with the Magen Microbiome kit also had increased levels of contamination. Other kits demonstrated contamination levels comparable to or lower than the reference kits (Figure 4C and Figure S8). However, it should be taken into account that some OTUs present in “kitomes” can still overlap with the OTUs present in environmental samples.

### 2.7. Effects of DNA Extraction Kits on the Composition of Bacterial Communities

**Figure 5:**
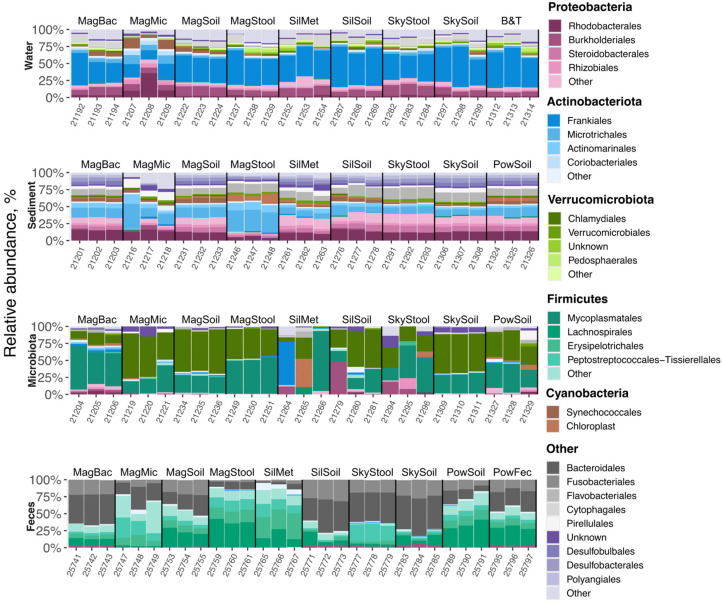
Effect of DNA extraction kit on microbiome composition and diversity of microorganisms. Relative abundance of bacterial orders in four sample types processed with different tested DNA extraction kits after contamination removal with Decontam. Bacterial orders with relative abundances > 1% are shown. Data for three technical replicates are shown. Numbers indicate internal labels for different replicates (BioProject ID PRJNA1183575).

**Figure 6:**
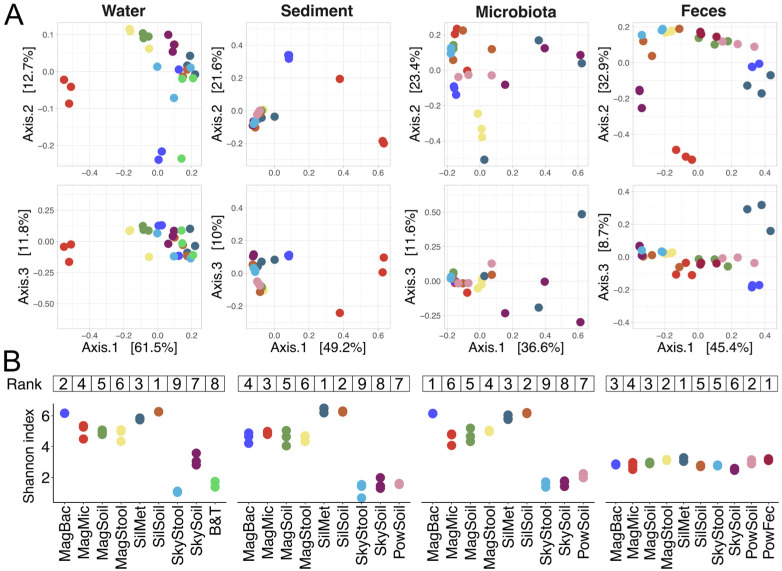
Effects of DNA extraction kit on beta- and alpha-diversity of microbiomes. (**A**)—PCoA of Bray–Curtis dissimilarity of samples processed with different DNA extraction kits, projections of principal components 1 and 2 (above) and 1 and 3 (below) are shown. Color coding is identical to panel (**B**). (**B**) Shannon index values of samples processed with different tested DNA extraction kits. Data for three technical replicates are shown. Numbers above represent kit ranking. Lower rank indicates higher Shannon index.

Next, we removed contaminant OTUs from the obtained datasets and inspected the effects of different DNA extraction kits on the composition of microbiomes. The composition of marine sediment and freshwater microbiomes was visibly more stable than that of the microbiota and fecal samples (Figure 5). A strong variation in the relative abundances of the bacterial taxa observed for the gut microbiota correlates with the increased contamination levels in these samples (Figure 4C). Samples processed with the Magen Microbiome kit showed inconsistent composition for all sample types (Figure 5) and were distant from other conditions (except for the gut microbiota samples) according to the beta-diversity metrics (Figure 6A), which may indicate a significant distortion of the bacterial community composition with this kit. Additionally, two replicates of freshwater samples processed with the Magen Bacterial kit and a single replicate produced by the Blood & Tissue kit had a deviated microbiome composition, while the other kits showed consistent community compositions. Similarly, marine sediment samples processed with the Magen Bacterial kit had a different composition from the samples produced by other kits (except for the Magen Microbiome discussed above), including the reference kit: these samples have an increased abundance of Acidimicrobiales and depleted Rhodobacterales. Gut microbiota samples processed with Magen Stool (increased Mycoplasmatales and decreased Chlamydiales), Sileks Metagenome, SkyGen Soil (highly inconsistent composition of replicates), and, to a lesser extent, Sileks Soil, deviated from a cluster of samples produced by other kits, including the reference kit (Figure 5 and Figure 6A). Fecal samples processed with SkyGen Soil (increased Erysipelotrichales, decreased Peptostreptococcales), Sileks Metagenome, and Magen Bacterial (both increased Eubacteriales and reduced Bacteroidales and Fusobacteriales) deviated from other samples by a beta-diversity metric (Figure 5 and Figure 6A).

Overall, our results confirm that the DNA extraction procedure could significantly affect the representation of OTU abundance, and for each sample type, we identified kits producing a similar microbiome composition to that obtained with a reference kit.

### 2.8. Effects of DNA Extraction Kits on the Detected Alpha-Diversity of Bacterial Communities

To assess the bacterial community sampling depth by different DNA extraction kits, we calculated an alpha-diversity metric, the Shannon index, for decontaminated microbiomes. Though we do not know the real compositions of these communities, we assume that a higher Shannon index indicates better sampling.

Multiple kits showed higher Shannon index values for freshwater, marine sediments, and *M. gigas* gut microbiota samples than the reference kits (Figure 6B). Particularly, Sileks Metagenome and Sileks Soil kits had the highest values for these sample types, and Magen Bacterial also performed well for freshwater and microbiota samples. In contrast, SkyGen Stool and SkyGen Soil had decreased Shannon index values with freshwater, marine sediments, and microbiota samples. Shannon index values obtained for the fecal samples were more homogeneous, and the reference PowerSoil and PowerFecal kits, together with the Magen Bacterial kit, slightly outperformed the other kits (Figure 6B).

Considering the Shannon index and contamination levels, the Sileks Metagenome and Sileks Soil kits preserved the highest diversity with the lowest contamination (except for a single replicate of a microbiota sample for the Sileks Metagenome kit) of the tested microbiomes. Magen Bacterial, Magen Soil, and Magen Stool also performed well in these benchmarks (Figure S9).

### 2.9. Technical Reproducibility of DNA Extraction Kits

As we noticed in the previous section, several kits (e.g., Magen Microbiome) produced inconsistent microbiome compositions across technical replicates. To quantify variability across the technical replicates, we used a reproducibility level—a fraction of OTUs found in all three replicates in the total number of OTUs found at least in one replicate [42].

**Figure 7:**
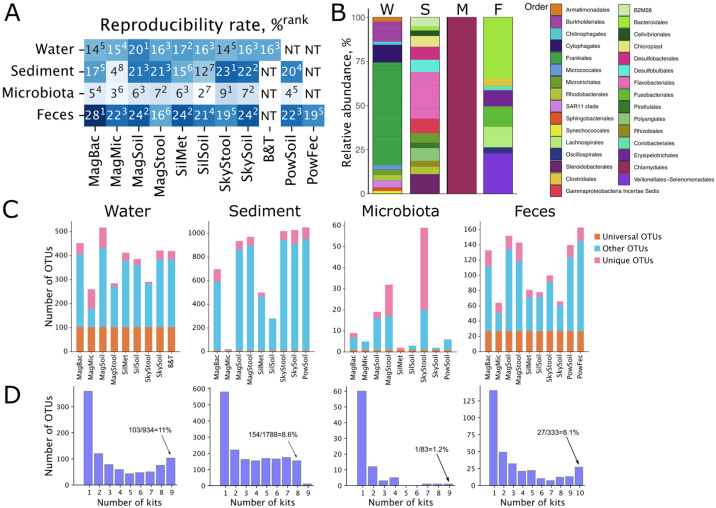
Effects of DNA extraction kits on the reproducibility of microbiome composition. (**A**) Reproducibility levels across four sample types processed with different DNA extraction kits. NT—not tested. Kit rank is shown in superscript. Lower rank indicates higher reproducibility rate. (**B**) Relative abundance of universal OTUs found across all sample types. Mean abundance for each of the following sample types is shown: water samples (W), sediment samples (S), microbiota samples (M), and fecal samples (F). (**C**) Stacked bar plots representing fractions of OTUs shared between all tested DNA extraction kits (universal OTUs, orange), OTUs found by just one kit (unique OTUs, pink), and OTUs found in DNA extracted by more than one but not all kits (other OTUs, light blue) for different sample types. (**D**) Numbers of OTUs shared between different numbers of DNA extraction kits tested; from 1—OTU was found in a single sample processed with a single kit, to 10—OTU was found in all samples processed with all tested kits.

For freshwater samples, the Magen Soil kit was the most consistent, with a reproducibility rate higher than that of the reference Blood & Tissue kit (Figure 7A and Figure S10). With this sample type, all kits were able to capture a substantial fraction of a microbiome, which was reflected by a relatively high number of OTUs shared among all DNA extraction conditions—103 universal OTUs were detected (11% of all OTUs) (Figure 7C,D). These OTUs were dominated by Frankiales, Burkholderiales, and Cytophagales (Figure 5 and Figure 7B). For marine sediment samples, SkyGen Soil showed better performance than the reference kit, and Magen Microbiome had an unexpectedly low rate, significantly lagging behind other kits (Figure 7A and Figure S10). The poor performance of the latter kit resulted in a low fraction of universal OTUs (Figure 7C,D). If we were to remove this kit from the analysis, the other kits shared 154 universal OTUs (8.6% of all OTUs) with dominating OTUs belonging to Acidimicrobiales, Actinomarinales, Rhodobacterales, Synechococcales, and Flavobacteriales (Figure 7B), similarly to what was observed for individual samples (Figure 5). Consistent with previous observations (Figure 5 and [42]), *M. gigas* gut microbiota samples had much lower reproducibility levels, which might be caused by the lower DNA yields, lower DNA quality, and higher contamination levels obtained for these samples or the innate heterogeneity of the sample (Figure 7A and Figure S10). Gut microbiota samples shared only 1 OTU (1.2% of all OTUs) among all DNA extraction conditions, indicating a dramatic effect of the DNA extraction kit on the microbiome composition (Figure 7C,D). This OTU was classified as Chlamydiales (Figure 7B), which dominated individual samples (Figure 5). Despite that, multiple DNA extraction kits (especially, SkyGen Soil and Sileks Metagenome) showed higher reproducibility rates compared to the reference Power Soil kit (Figure 7A). Similarly to the freshwater samples, fecal samples demonstrated high reproducibility rates and shared 27 universal OTUs (8.1% of all OTUs) among all DNA extraction conditions. Universal OTUs were dominated by Bacteroidales, Fusobacteriales, and Veillonellales (Figure 7B). Magen Bacterial, Magen Soil, and SkyGen Soil extracted DNA with better reproducibility than the reference kits (Figure 7A and Figure S10).

Overall, the Magen Soil kit showed the highest consistency for the freshwater samples, outperforming the reference Blood&Tissue kit. In marine sediment samples, the SkyGen Soil kit outperformed the others, while the Magen Microbiome kit performed poorly. Samples of *M. gigas* gut microbiota exhibited low overall reproducibility, with only one shared OTU across kits and the best reproducibility percentage of 9% for the SkyGen Stool kit. In contrast, the fecal samples showed high overall reproducibility, with several kits (notably Magen Bacterial, Magen Soil, and SkyGen Soil) surpassing the reference kits in consistency between replicates.

## 3. Discussion

Current knowledge of microbial abundance and diversity in Earth’s ecosystems relies on metagenomic approaches. However, these analyses can be significantly biased by the DNA extraction methodology employed, as different purification protocols exhibit varying efficiencies across taxonomic groups. In addition, the selection of extraction kits affects multiple DNA quality parameters that determine suitability for both short- and long-read sequencing. Finally, the time and labor costs associated with each protocol must be considered when designing metagenomic studies.

Previously, we systematically benchmarked commonly used DNA extraction kits using environmental samples that are important for marine metagenomics. We identified that Qiagen kits (Blood&Tissue for water samples and PowerSoil/PowerFecal for sediments and microbiota samples) provided high-quality DNA suitable for reproducible 16S sequencing [42]. However, these kits tend to be among the most expensive options available on the market. Here, we set the goal of identifying alternative, more affordable DNA extraction kits that could reproducibly extract high-quality DNA suitable for metagenomic studies.

We tested several kits dedicated to DNA extraction from environmental and fecal samples manufactured by Magen (China), Sileks (Russia), and SkyGen (Russia). To assess these kits, we considered three groups of parameters: accessibility—cost and simplicity of usage; DNA quality—DNA yield and DNA integrity (DIN), presence of PCR inhibitors and admixture of eukaryotic DNA; suitability of a kit for 16S metagenomics—contamination of samples during DNA extraction, alpha-diversity (the Shannon index), and reproducibility of a microbiome composition. To compare the selected kits, we used four sample types: freshwater, marine sediments, *M. gigas* gut microbiota (representing a microbiome associated with a marine invertebrate), and commonly studied mammalian fecal microbiota.

**Figure 8:**
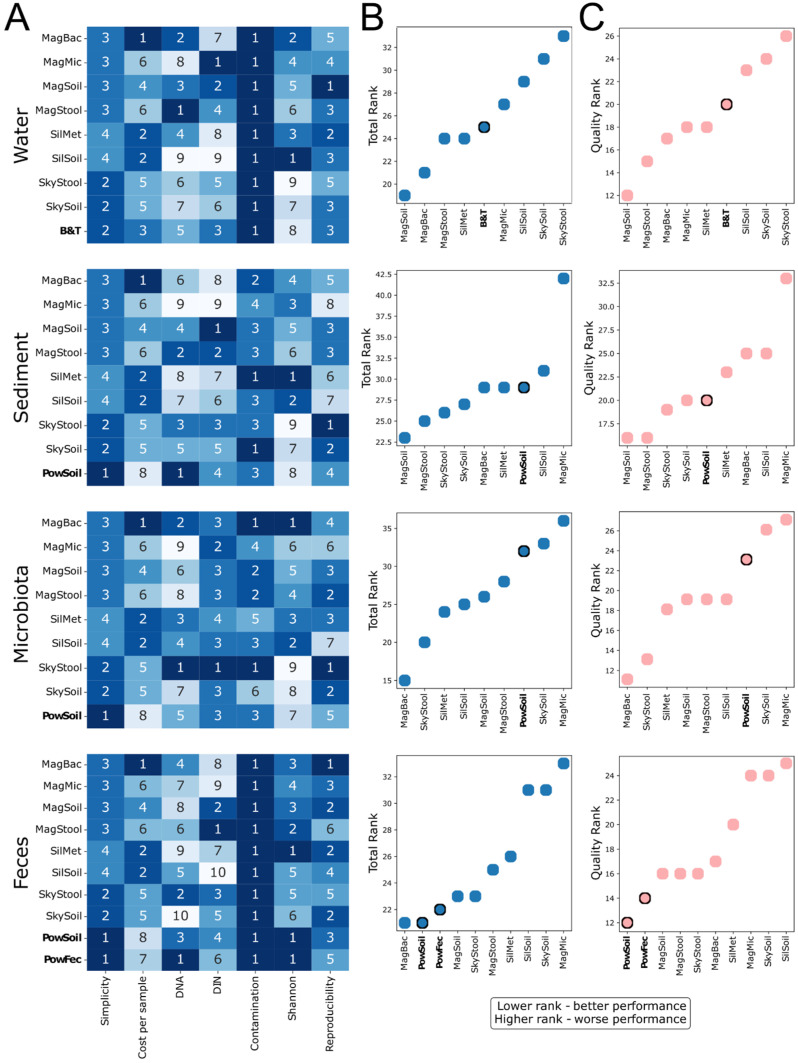
Resulting ranking matrix of the tested DNA extraction kits applied for different sample types. (**A**) Heatmaps representing kit ranks obtained in different tests for different sample types. (**B**) Total aggregated sorted kit ranks for different sample types. (**C**) Aggregated and sorted kit ranks excluding the simplicity and cost-per-sample ranks. Reference kits are highlighted in bold. For each evaluated parameter, 8 tested kits and reference kits were grouped and ranked for their performance. The scale for each parameter reflects the number of groups, and lower ranks indicate better performance.

Considering the sum of assessed parameters, we identified multiple alternative kits that performed comparably or better than selected reference kits for most sample types (Figure 8). For freshwater samples, Magen Soil and Magen Bacterial kits performed the best and significantly surpassed the reference Blood&Tissue DNA extraction protocol (Figure 8B). The Magen Soil kit showed no DNA integrity issues and can therefore be recommended for long-read sequencing of freshwater samples. The Magen Bacterial kit, however, produced DNA with reduced integrity, which might affect its application for shotgun and, particularly, long-read sequencing. SkyGen kits, Sileks Soil, and Magen Microbiome kits, in contrast, lagged behind the other kits and therefore could not be recommended for this type of sample (Figure 8A). For marine sediment samples, Magen Soil and Magen Stool significantly outperformed the reference PowerSoil kit (Figure 8B). Other kits performed similarly, except for Magen Microbiome, which was indicated as an outsider by its DNA yield, DNA integrity, and microbiome composition reproducibility parameters (Figure 8A). Therefore, the Magen Microbiome kit cannot be recommended for shotgun or long-read sequencing due to its low DNA integrity, whereas the Magen Soil and Magen Stool kits outperformed the other kits and can be considered suitable for this sample type. For *M. gigas* gut microbiota samples, Magen Bacterial and SkyGen Stool significantly outperformed the other kits. The reference PowerSoil kits surpassed only the Magen Microbiome and SkyGen Soil kits, which could not be recommended for this sample type (Figure 8B). The low DIN values for the SkyGen Soil kit indicate degraded DNA, while the Magen Microbiome kit produced slightly higher DNA integrity, which may be relevant when considering its use in long-read sequencing applications. For fecal samples, the reference kits PowerSoil and PowerFecal shared the top performance with the Magen Bacterial and Magen Soil kits (Figure 8B). The latter kits, therefore, can be considered as more affordable alternatives to Qiagen kits. The Magen Bacterial kit generated degraded DNA for fecal samples and should be used with caution if samples are selected for long-read sequencing (Figure 8A). In general, for any kit tested, the DIN values for fecal samples were substantially lower than the recommended values of above 7. This could reflect the freezing and storage conditions of these samples, which decreased the quality of the DNA [54]. Considering this limitation, the Magen Soil kit can be recommended as the variant producing the best DIN value for the fecal material. The Magen Microbiome and SkyGen Soil kits were among the outliers and could not be recommended for DNA extraction from fecal samples.

After correcting for the kits’ simplicity and cost, the proposed ranking remained mostly unchanged (Figure 8C). The evaluated DNA parameters could not be considered equal for the selection of the kit, and we recommend aligning these values with the specific goals of the project (shotgun or 16S sequencing). Taken together, Magen Bacterial and Magen Soil kits performed relatively better than the other kits, including the reference kits, and were highlighted among the top-performing kits for three out of four sample types.

Our comprehensive benchmarking of commercial DNA extraction kits revealed that several cost-effective alternatives to the widely used Qiagen kits can yield high-quality DNA suitable for 16S amplicon sequencing applications for metagenomics across diverse environmental sample types. We evaluated kits from Magen (China), SkyGen (Russia), and Sileks (Russia) based on a wide range of metrics, including DNA yield, integrity, purity, inhibitor presence, contamination levels, alpha-diversity, and reproducibility, as well as processing complexity and cost.

Across all metrics, the Magen Soil and Magen Bacterial kits consistently performed well, often surpassing the reference kits in microbiome fidelity, reproducibility, and microbial DNA selectivity. However, the Magen Bacterial kit occasionally yielded more fragmented DNA, which may limit its use for long-read sequencing. SkyGen Stool also showed a strong performance for host-associated microbiota, while Sileks Soil and Sileks Metagenome stood out in preserving microbial diversity with minimal contamination, particularly in the sediment samples. Notably, the Magen Microbiome kit demonstrated poor reproducibility, low yield, and high contamination in multiple contexts, making it unsuitable for metagenomic analyses.

Our findings underscore the importance of selecting DNA extraction kits based on the specific sample type and research goals. This study provides a practical guide for selecting optimal DNA extraction protocols that balance cost, labor, and downstream data quality in metagenomics workflows.

It should be noted that our work does not account for possible sample heterogeneity. To achieve uniformity of purification procedures, we treated the homogeneous samples (pooled gut microbiota or fecal material, and water/sediment samples from the same batch) with different kits. However, biological samples obtained from different individuals or collected at different locations will demonstrate enormous variability in these parameters, contributing to the quality of the purified DNA. Thus, while the current study represents an approximation of the efficiency of each procedure for a particular sample type, more comprehensive research is needed to generalize our conclusions. We should also note that possible batch-to-batch variability of the tested kits and their different production dates represent other potential limitations of the generalization of our conclusions.

## 4. Materials and Methods

### 4.1. DNA Purification Kits

We selected eight commercially available DNA purification kits, considering their costs and wide availability across the markets of scientific reagents in China and Russia. As a reference, we used three previously tested kits (Qiagen PowerSoil, PowerFecal and Blood&Tissue). All DNA extraction steps were performed according to the manufacturer’s recommendations. As a recommended part of some protocols, the DNase I treatment step was applied to all sample types, and it should be noted that this procedure could affect the community composition. At all stages where water was required, we used sterile nuclease-free water (B1500L, NEB). If the bead beating step was necessary, we used TissueLyser LT (Qiagen, Hilden, Germany) and treated samples at conditions of 50 Hz for 10 min, unless another time was specified in the protocol. If necessary, we specifically mention some minor changes introduced into the protocol. The expiration date had not expired for any kits used. A general description of each kit and its short common name is provided below.

#### 4.1.1. DNeasy Blood and Tissue Kit (#69504 Qiagen, Hilden, Germany) = B&T (Blood&Tissue) Kit

After cell lysis and protein degradation in the presence of Proteinase K, the sample is loaded onto the DNeasy Mini spin column. DNA is washed twice and eluted from the silica membrane.

#### 4.1.2. DNeasy PowerSoil Pro Kit (#47016 Qiagen, Hilden, Germany) = PowerSoil Kit

The protocol starts with mechano-chemical cell disruption through bead beating in a lysis solution. In this kit, Inhibitor Removal Technology is applied, which should help in the removal of organic and inorganic materials such as humic acids, cell debris, and proteins that could interfere with downstream PCR applications. After that, the sample is loaded onto the MB Spin Column with a silica membrane. DNA is washed twice and eluted from the silica membrane.

#### 4.1.3. QIAamp PowerFecal Pro DNA Kit (#51804-50 Qiagen, Hilden, Germany) = PowerFecal Kit

In the first step, cells are disrupted mechanically (by bead beating) and chemically (with lysis buffer). Then the inhibitors are removed from the sample by using Inhibitor Removal Technology^®^ to prevent interference with the downstream enzymatic reactions, such as PCR. Finally, DNA is captured from the supernatant by using an MB Spin column, washed twice, and eluted from the silica membrane.

#### 4.1.4. HiPure Stool DNA Kit (#IVD3141 Magen, Guangzhou, China) = Magen Stool Kit (MagStool)

In this kit, the sample is treated in a detergent-containing buffer that lyses bacteria, yeast, and fungi, followed by phenol extraction that removes humic acid, proteins, polysaccharides, and other organic impurities. After chemical–mechanical cell lysis and protein removal, the DNA is precipitated with ethanol, and the sample is transferred into the HiPure DNA Mini Column II. After three washes, DNA is eluted from the silica membrane with a low ionic strength buffer.

#### 4.1.5. HiPure MicroBiome DNA Kit (#D314802, Magen, Guangzhou, China) = Magen Microbiome Kit (MagMic)

This kit is based on silica column purification. Cells are incubated in a buffer, treated with DNase I to remove extracellular DNA, followed by mechanical lysis and treatment with proteinase K. Then sample is transferred into the HiPure DNA Mini Column I. Washed twice, DNA is eluted with a low ionic strength buffer.

#### 4.1.6. HiPure Soil DNA Kit (#D314202, Magen, Guangzhou, China) = Magen Soil Kit (MagSoil)

Samples are homogenized and then lysed in a detergent-containing buffer that lyses bacteria, yeast, and fungi. Then, humic acid, proteins, polysaccharides, and other contaminants are removed with a special solution. DNA is transferred to the HiPure DNA Mini Column II and washed three times. Finally, DNA is eluted in a low ionic strength buffer.

#### 4.1.7. HiPure Bacterial DNA Kit (#D314602, Magen, Guangzhou, China) = Magen Bacterial Kit (MagBac)

This kit is based on silica column purification. The sample is lysed and digested with a lysis buffer supplemented with protease. The DNA is transferred to the HiPure DNA Mini Column I, and washed twice, followed by elution with a low ionic strength buffer.

#### 4.1.8. Nucleic Acid Extraction for Metagenomic Analysis (#KIMB0100, Sileks, Moscow, Russia) = Sileks Metagenomic Kit (SilMet)

This kit applies selective disruption of the eukaryotic cells, followed by treatment with an endonuclease to remove host DNA. This approach implies enrichment of bacterial and viral DNA in a sample. After microbial cell lysis, DNA binds to magnetic beads, followed by washing and elution with a low ionic strength buffer.

#### 4.1.9. SileksMagNA Kit (#KIRSL0100, Sileks, Moscow, Russia) = Sileks Soil Kit (SilSoil)

This kit is intended for the isolation of DNA and RNA from soil samples. The main steps of the protocol include cell lysis, homogenization, DNA binding to magnetic beads, washing, and elution.

#### 4.1.10. SKYAmp Soil DNA Kit (#EDC336, SkyGen, Moscow, Russia) = SkyGen Soil Kit (SkySoil)

This kit relies on specific buffers intended to remove humic acid and other organic impurities from soil DNA samples. Cells are incubated in a chemical lysis solution, followed by homogenization and contaminant removal treatment. The DNA is transferred to a silica-membrane spin column, washed twice, and eluted with a low ionic strength buffer.

#### 4.1.11. SKYAmp Stool DNA (#EDC328, SkyGen, Moscow, Russia) = SkyGen Stool Kit (SkyStool)

Similarly to the previous example, this kit should remove organic impurities, e.g., humic acid. The first steps include chemical–mechanical disruption of the cells. The sample is treated with RNAse A and an organic contaminant removal buffer. The DNA is transferred to a silica-membrane spin column, washed twice, and eluted with a low ionic strength buffer.

### 4.2. Samples Collection

#### 4.2.1. Freshwater Samples

Freshwater samples were collected from Skolkovo pond (Moscow, 55.69491 N, 37.35393 E) in August 2023 (Figure S1, Table 2). For each sample, 800 mL of water from the same batch was filtered through the 0.22 μm Sterivex unit (Merck-Millipore, Darmstadt, Germany) with a peristaltic pump. 800 mL of Milli-Q water was filtered as a negative control in parallel to capture and investigate the “splashome”. Filters were subsequently stored at −20 °C until processing.

#### 4.2.2. Gut Microbiota Samples

Six individuals of *Magallana gigas* (also known as *Crassostrea gigas* (NCBI:txid29159)) were collected in Melkovodnaya Bay (Vladivostok, 43.2052 N, 131.9263 E) in June 2023 (Figure S1, Table 2). Digestive tracts were cut with a sterilized scalpel blade on the day of collection and stored at −20 °C until processing.

#### 4.2.3. Sediment Samples

The sea sediment sample was collected during the period of low tide at the littoral zone of the Barents Sea (Ura Bay, 69.2951 N, 32.8372 E) in August 2023 (Figure S1, Table 2). The material was hand-collected with a scoop from a sediment horizon of 0–5 cm at one spot and placed in one 50 mL plastic tube. The sample was sterile packed, transferred, and stored at −20 °C until further processing.

#### 4.2.4. Mammalian Fecal Samples

Mammalian fecal samples were collected from a dog of the Welsh Corgi breed (*Canis lupus*) in Moscow (55.7130 N, 36.9155 E) in January 2024 (Figure S1, Table 2). The material was hand-collected and placed in one sterile medical container. Sample was sterile packed, transferred and stored at −20 °C until further processing.

### 4.3. DNA Extraction

#### 4.3.1. Freshwater Samples

Sterivex filter membrane was removed from the cover as described previously [55]. The membrane was transferred into a sterile single-use Petri dish with a cell-coated surface facing up and cut into small pieces. Membrane fragments were transferred into the sterile 2 mL microcentrifuge tube. Before further processing, filter fragments were incubated in 200 μL of the lysis buffer STET (50 mM TrisHCl, pH = 8.0; 50 mM EDTA; 5% Triton-X100; NaCl—200 mM; freshly supplied with 10 mg/mL lysozyme) at 37 °C for 30 min in a heating block with shaking at 600 rpm. After incubation, all liquid was collected for downstream processing in accordance with the manufacturer’s protocols. Three Sterivex filter membranes (Merck-Millipore, Darmstadt, Germany) were used for each kit tested. All water samples were obtained from the same batch and three filters were used per each condition tested.

#### 4.3.2. Gut Microbiota Samples

Digestive tracts from six *M. gigas* individuals were thawed, pooled, and homogenized for 15 min at 50 Hz using TissueLyser LT in the Tissue Disruption Tube (QIAamp Fast DNA Tissue Kit (Qiagen)), containing a single stainless-steel bead. Any other tissue homogenization method can be applied as this step. The resulting homogenate was split into aliquots of ~300 mg (three replicates for each kit tested) and aliquots were directly mixed with the kit lysis solution. DNA was isolated in accordance with the manufacturer’s protocols. All samples treated in this work were obtained from the same pooled homogenate.

#### 4.3.3. Sediment Samples

Sediment material for all kits was taken from the same tube in technical replicates, after rigorous mixing that decreases material heterogeneity. 1 g of thawed sediment was used per replicate. Material was directly mixed with the kit lysis solution. DNA was isolated in accordance with protocols recommended by manufacturers. All sediment samples were obtained from the same batch and three replicates were used per each condition tested.

#### 4.3.4. Mammalian Fecal Samples

Fecal material for all kits was taken from the same tube in three replicates The sample was thoroughly mixed to ensure uniformity and aliquots equivalent to 300 mg of dry weight were taken for DNA extraction. DNA was isolated in accordance with protocols recommended by manufacturers. All samples treated in this work were obtained from the same pooled homogenate.

#### 4.3.5. “Kitomes” and “Splashomes”

For each kit, two types of negative controls were prepared in three replicates. For No-input negative control or “kitome”, no starting material was added, and DNA was extracted directly from the buffers contained in the kit. For mQ negative control or “splashome”, laboratory mQ water was purified in two steps using the Barnstead Pacific TII (Thermo Scientific, Waltham, MA, USA) and Simplicity (Millipore, Darmstadt, Germany) filtration systems. 800 mL of mQ was filtered through Sterivex filter units, and DNA was extracted from filter membranes as described for Water samples.

### 4.4. DNA Quantification and Quality Assessment

DNA concentration was measured using the Lumiprobe QuDye BR kit or QuDye HS kit on the Qubit 4.0 Fluorometer (Invitrogen, Carlsbad, CA, USA). All DNA samples were eluted with 60 μL of elution buffer provided in the corresponding kit so the total amount can be directly compared within one sample type. The detection limit of DNA concentration in the sample for the QuDye HS Kit is 0.1 ng/μL. Thus, samples with no DNA detected with this assay should contain less than 6 ng of total DNA. We consider samples with DNA yield above 1500 ng as suitable for shotgun DNA sequencing.

DNA purity was assessed by measuring the ratios of absorbance at 260 nm to 230 nm (260/230) and 260 nm to 280 nm (260/280) using the NanoDrop1000 spectrophotometer (Thermo Scientific, Waltham, MA, USA). Samples with a 260/230 ratio between ∼2.0–2.2 and 260/280 ∼1.7–2.0 are assumed as “pure”.

The integrity of genomic DNA was assessed using Agilent TypeStation 4150 (Agilent Technologies, Santa Clara, CA, USA) with Genomic DNA ScreenTape System according to the manufacturer’s instructions. High concentration samples require 1 µL of sample for analysis. For samples with DNA concentrations lower than 7 ng/μL, the samples have been dried using a HyperVAC concentrator (Gyrozen, Republic of Korea) in order to increase the nucleic acid concentration. Samples with DIN above 7.0 were assumed as “high-quality” and acceptable for long-read sequencing.

### 4.5. PCR Amplification

16S rRNA V3-V4 region was amplified using universal Illumina V3/V4 PCR primers (Table 3). Each PCR reaction contained 1 μL of DNA, 0.25 μL of forward and reverse primer (final concentration of 0.5 mM), 5 μL of Phusion High-Fidelity PCR Master Mix with HF Buffer (NEB) and 3 μL of nuclease-free water (NEB) for final reaction volume of 10 μL. PCR conditions were 98 °C for 30 s, followed by 25 cycles of 98 °C for 10 s, 55 °C for 20 s, and 72 °C for 30 s, with a final extension time of 5 min at 72 °C. Amplicons were visualized on a 1% agarose gel containing 0.25 μg/μL ethidium bromide running in 1x Tris-EDTA buffer at 100 V with a target product size in a range of 400–500 bp. Products of successful amplification were clearly visible on gel, otherwise the initial DNA sample was diluted 10 or 100 times for PCR re-examination. While we used ethidium bromide staining for all DNA visualization procedures, alternative dyes like SYBR Safe are applicable and could be recommended for a safer performance.

### 4.6. Quantitative Real-Time PCR

To determine the ratio of bacterial DNA to co-extracted eukaryotic DNA, quantitative real-time polymerase chain reaction (qPCR) was performed targeting different variable regions of the 16S ribosomal RNA (rRNA) and 18S rRNA genes using universal primers (Table 3). Due to the presence of potential DNA inhibitors in many samples, additional dilution was required to achieve robust amplification. However, the same DNA sample was used for both 16S and 18S amplifications.

qPCR reactions were carried out in technical triplicate in 96-well optical plates using the QuantStudio 3 Real-Time PCR system (Applied Biosystems, Foster, CA, USA). Each reaction was performed in 10 μL and included 1 μL of template DNA, 2 μL of 5x qPCR mix-HS SYBR + LowROX (Evrogen, Moscow, Russia), and 0.5 μL each of forward and reverse primers at a final concentration of 0.5 mM. The qPCR protocol consisted of an initial denaturation step at 95 °C for 5 min, followed by 40 amplification cycles of 30 s at 95 °C, 20 s at 55 °C and 30 s at 72 °C. A final dissociation melt curve generation step was performed to verify the amplification specificity.

The load of eukaryotic DNA was calculated as the difference between the threshold cycle values (Ct) obtained for each sample using 16S- and 18S-specific primers. This calculation was performed by subtracting the Ct(18S) value from the Ct(16S) value.

### 4.7. 16S rRNA Libraries Sequencing and Data Analysis

Amplification of the V3–V4 region of 16S rRNA and library preparations were performed according to the Illumina manual [56]. The amplicon libraries were barcoded, pooled, and sequenced using NovaSeq 6000 (Illumina, San Diego, CA, USA), 2  ×  250 bp paired-end protocol at Evrogen (Moscow, Russia).

For 16S data analysis, a snakemake pipeline for paired-end 16S sequencing data processing was developed (https://github.com/chorzow/16S_PE/) [42]. Briefly, the quality of sequencing data was estimated using FastQC (Version 0.12.0), then reads were trimmed and filtered using Trimmomatic v0.39 [58] in paired-end mode with the following parameters: -phred 33 ILLUMINACLIP:2:30:10 SLIDINGWINDOW:4:15 HEADCROP:17 MINLEN:150. Forward and reverse reads that passed quality control were further processed with DADA2 pipeline v1.26.0 [53]. Resultant denoised, merged, and non-chimeric amplicon sequence variants (ASVs) were clustered using MMseqs2 v13.45111 [59] (coverage  >  0.95, identity  >  0.98), and representative sequences were further treated as operational taxonomic units (OTUs). OTUs were returned to DADA2, and taxonomy was assigned to OTUs using the SILVA SSU database v.138 [42,60]. Contamination was removed using R package decontam v. 1.13.0 [53] in the “either” mode with a threshold of 0.2. Principal coordinate analysis (PCoA), alpha-diversity and taxonomic analyses were performed with R packages config v0.3.2, here v1.0.1, dplyr v1.1.4, ggplot2 v3.5.0, ggsci v3.2.0, lemon v0.4.9, patchwork v1.2.0, microshades v1.13, phyloseq v1.46.0 [61], scales v1.3.0, jsonlite v2.0.0, reshape2 v1.4.4, cowplot v1.1.3, vegan v2.6.2. Shannon index, calculated with phyloseq, was used as a metric that incorporates the richness and dominance of OTUs in a community. Reproducibility analysis was performed using Python v3.11.

## Figures and Tables

**Table 1:** Description of DNA kits used in the study.

Kit	Lysis Method	DNA Isolation Method	Simplicity (1–5)	Cost per Package	Cost per Sample
Magen Microbiome (#D314802, Magen, Guangzhou, China)	Mechanical, chemical	Spin column	3	$348.00/50 preps	$6.69
Magen Bacterial (#D314602, Magen, Guangzhou, China)	Mechanical, chemical, enzymatic	Spin column	3	$149.00/50 preps	$2.98
Magen Stool (#IVD3141, Magen, Guangzhou, China)	Mechanical, chemical, heat	Spin column	3	$348.00/50 preps	$6.69
Magen Soil (#D314202, Magen, Guangzhou, China)	Mechanical, chemical, heat	Spin column	3	$249.00/50 preps	$4.98
Sileks Soil (#KIRSL0100, Sileks, Moscow, Russia)	Chemical, mechanical	Magnetic beads	4	$434.78/100 preps	$4.35
Sileks Metagenomic (#KIMB0100, Sileks, Moscow, Russia)	Chemical	Magnetic beads	4	$434.78/100 preps	$4.35
PowerSoil (#47016 Qiagen, Hilden, Germany)	Mechanical, chemical	Spin column	1	$490.00/50 preps	$9.8
PowerFecal (#51804-50, Qiagen, Hilden, Germany)	Mechanical, chemical	Spin column	1	$483.00/50 preps	$9.66
Blood&Tissue (#69504, Qiagen, Hilden, Germany)	Heat, chemical, enzymatic *	Spin column	2	$224.00/50 preps	$4.48
SkyGen Soil (#EDC336, SkyGen, Moscow, Russia)	Chemical, mechanical	Spin column	2	$323.91/50 preps	$6.48
SkyGen Stool (#EDC328, SkyGen, Moscow, Russia)	Chemical, mechanical	Spin column	2	$323.91/50 preps	$6.48

* Samples were treated with Lysozyme, as described in Materials and Methods.

**Table 2:** Information on location, coordinates, and collection date for sample types used in the study.

Sample Type	Location	Coordinates	Sample Collection Date
Freshwater	Skolkovo pond	N 55.6949, E 37.3539	7 August 2023
Marine sediments	Barents Sea	N 69.2951, E 32.8372	19 August 2023
Gut microbiota (*M. gigas*)	Japan sea (Vladivostok)	N 43.2052, E 131.9263	24 June 2023
Mammalian feces (*C. lupus*)	Moscow	N 55.7130, E 36.9155	31 January 2024

**Table 3:** Forward (F) and reverse (R) PCR primers used for PCR amplification for the detection of inhibitors and quantitative real-time PCR.

Gene	Primer Name	Direction	Sequence 5′-3′	Source
16S (V3–V4)	16S_341_F	F	TCGTCGGCAGCGTCAGATGTGTATAAGAGACAGCCTACGGGNGGCWGCAG	[56]
16S (V3–V4)	16S_785_R	R	GTCTCGTGGGCTCGGAGATGTGTATAAGAGACAGGACTAAACHVGGGTATCTAATCC	[56]
18S (V4)	18S_Reuk454FWD1	F	CCAGCAGCCGCGGTAATTCC	[57]
18S (V4)	18S_ReukREV1	R	ACTTTCGTTCTTGATTAA	[57]

## Data Availability

The original data presented in the study are openly available in NCBI repository at https://www.ncbi.nlm.nih.gov/bioproject under BioProject ID PRJNA1183575.

## Supplementary Materials

The following supporting information can be downloaded at: https://www.mdpi.com/article/10.3390/ijms262311616/s1.

## Abbreviations

The following abbreviations are used in this manuscript:

DNA	Deoxyribonucleic acid
PCR	Polymerase chain reaction
