# Supplementary material for: Benchmarking Cost-Effective DNA Extraction Kits for Diverse Metagenomic Samples
Source: Int J Mol Sci. 2025 Nov 30;26(23):11616. doi: 10.3390/ijms262311616 (PMC12692181; doi:10.3390/ijms262311616)
Supplement: Supplementary file 1 [file ijms-26-11616-s001.zip › ijms-3976803-supplementary.pdf]

Supplementary Materials

Supplementary Figures

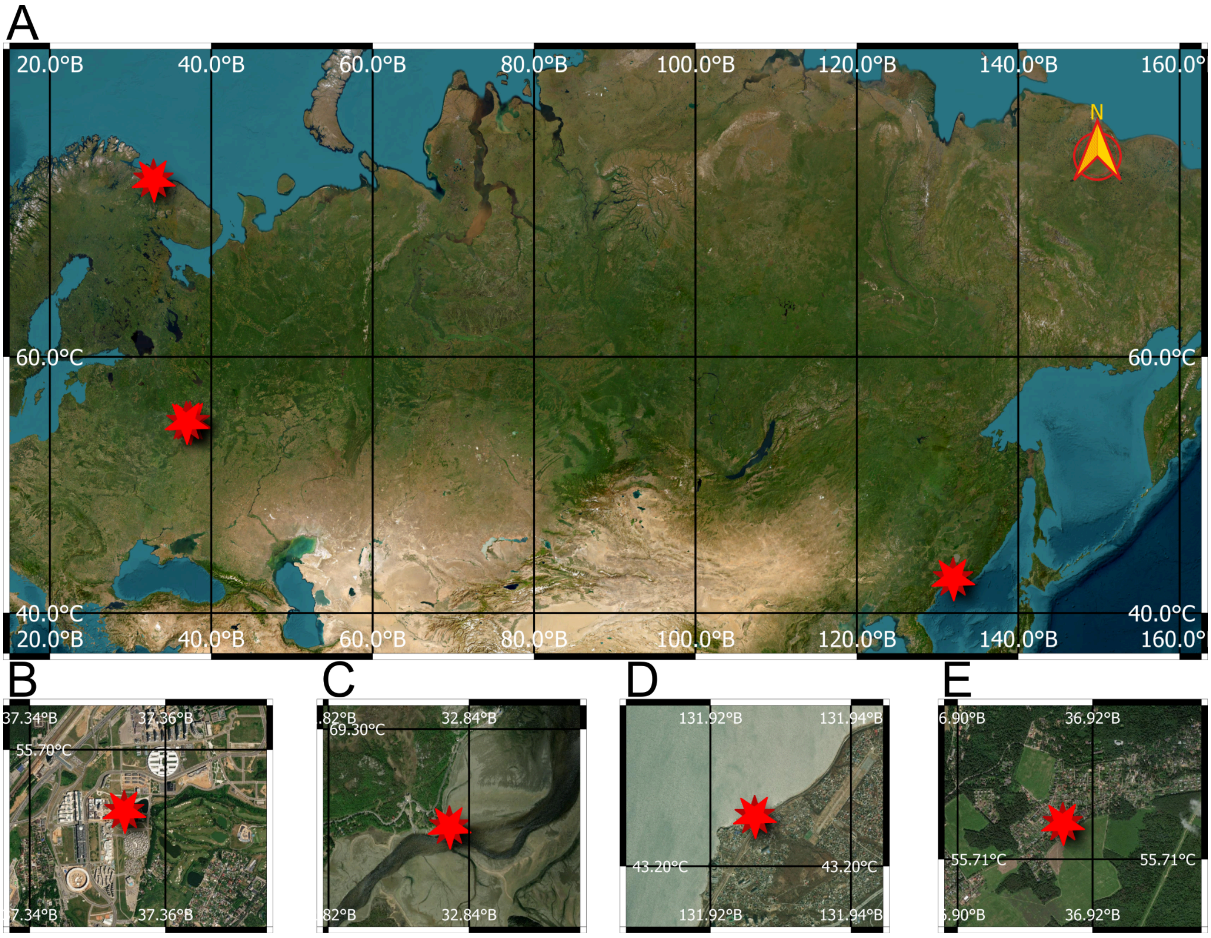

Figure S1. Geographical locations for sample collection spots. (**A**) — overview of all sample collection locations, (**B**)-(**E**) — sample collection locations for water (**B**), sediment (**C**), gut flora (**D**) and feces (**E**) samples, respectively. The map was prepared using the Open Source Geospatial Foundation Project (http://qgis.org).

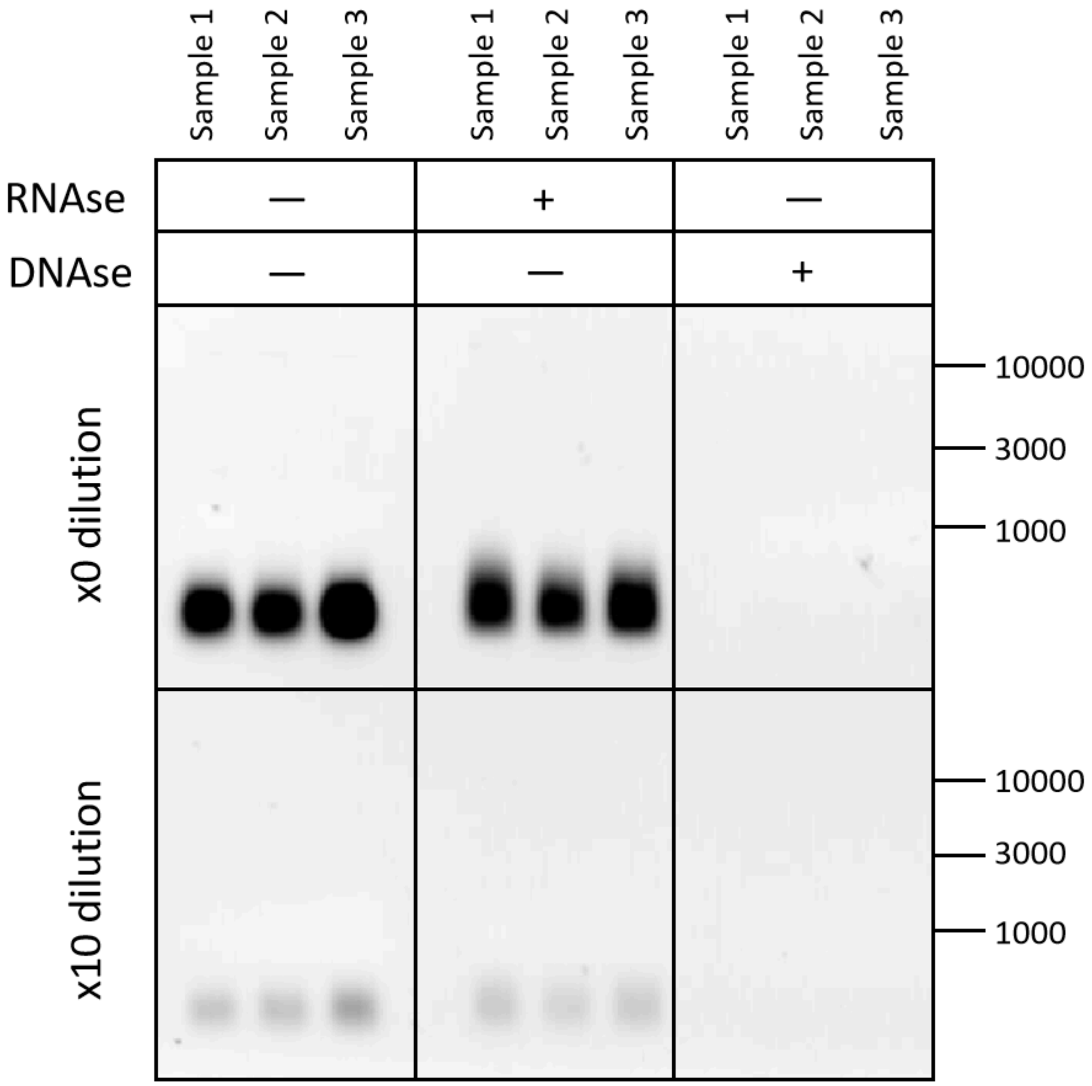

Figure S2. DNase and RNase treatment of M. gigas DNA samples isolated using the Skygen Stool kit.

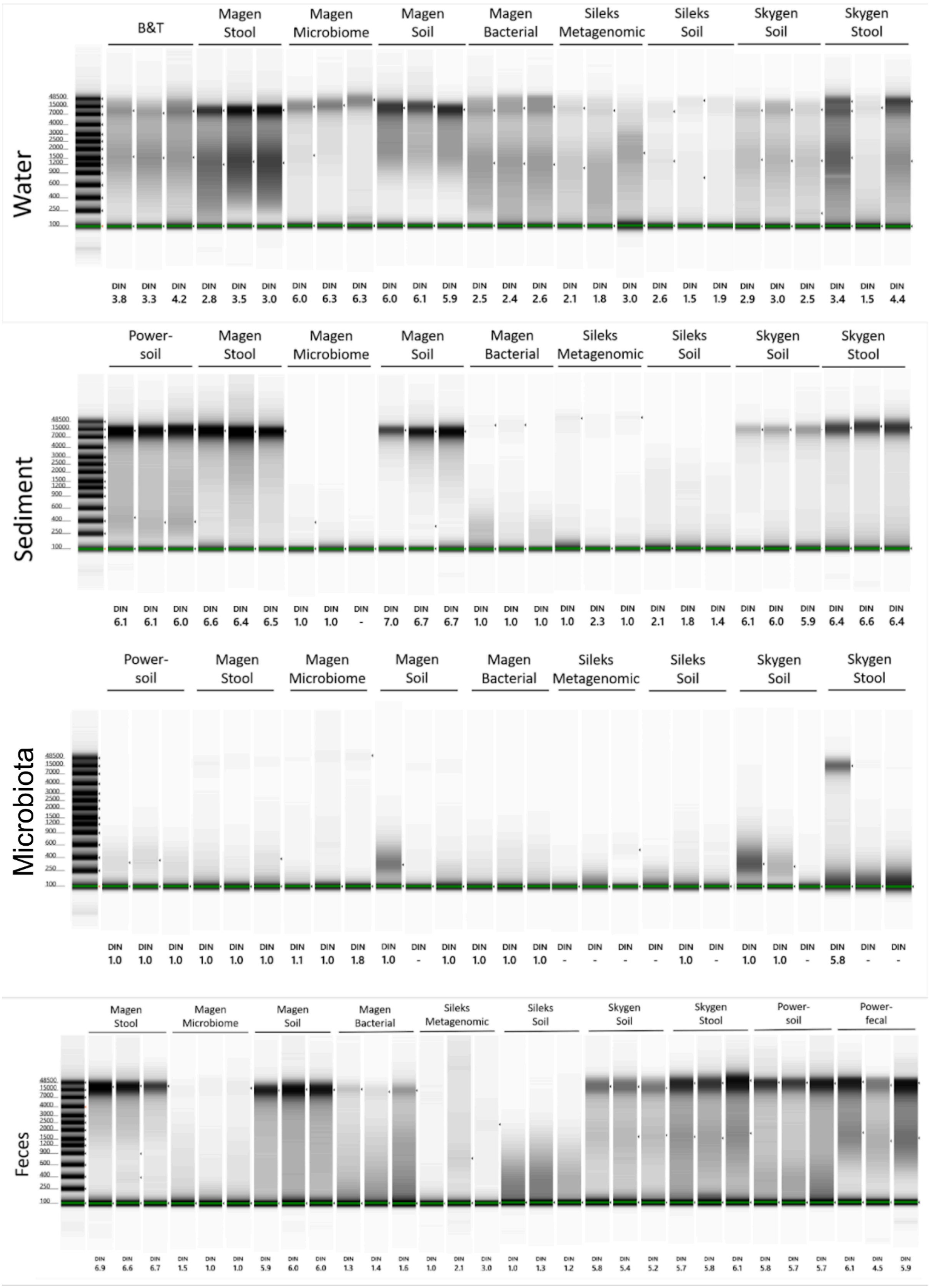

Figure S3. Capillary electrophoresis estimation of the DIN values performed on a TapeStation 4150 (Agilent) with Genomic DNA ScreenTape System for different kit-sample combinations.

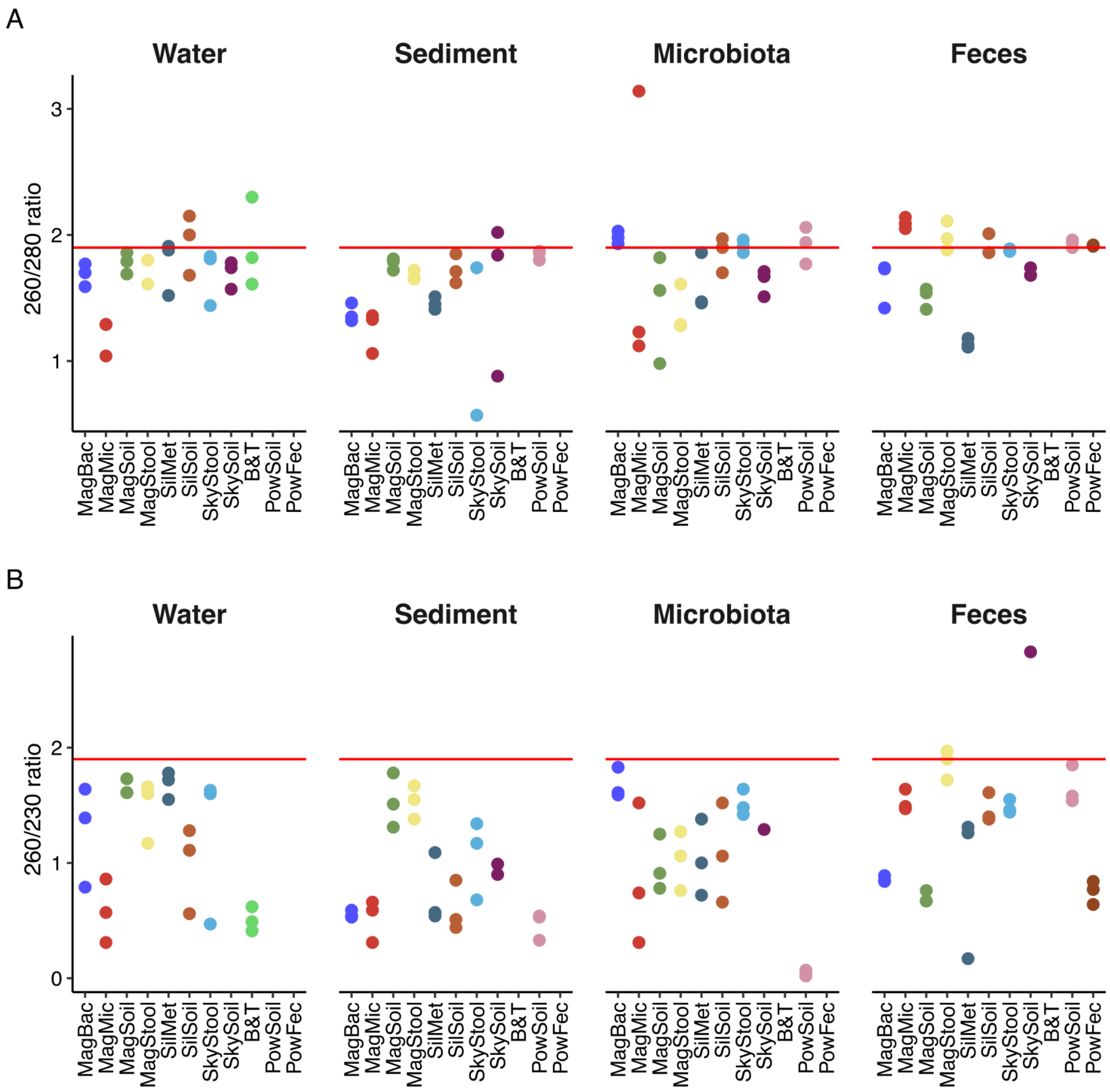

Figure S4. Purity of DNA samples extracted from four sample types with different kits tested assessed by 260/280 nm ratio (**A**) and 260/230 nm ratio (**B**) absorption ratios. Red line at 1.8 indicates a "high-quality" threshold. Data for three technical replicates are shown.

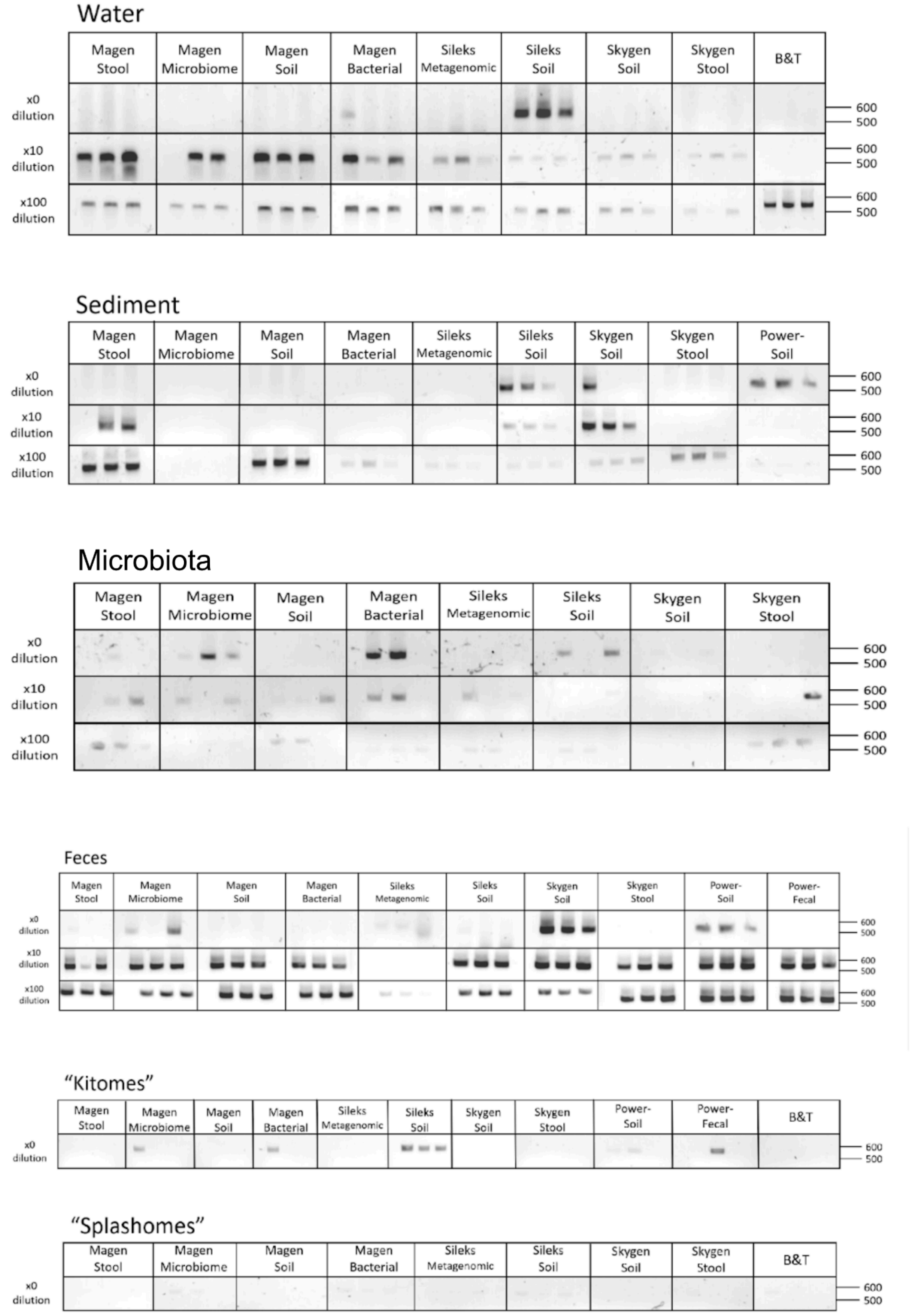

Figure S5. Results of the V3-V4 16S rRNA gene fragment PCR for DNA samples and control samples ("kitomes" and "splashomes") obtained with different kits. PCR was performed with non-diluted, 10- fold and 100-fold diluted input DNA. Molecular weight markers are shown on the right.

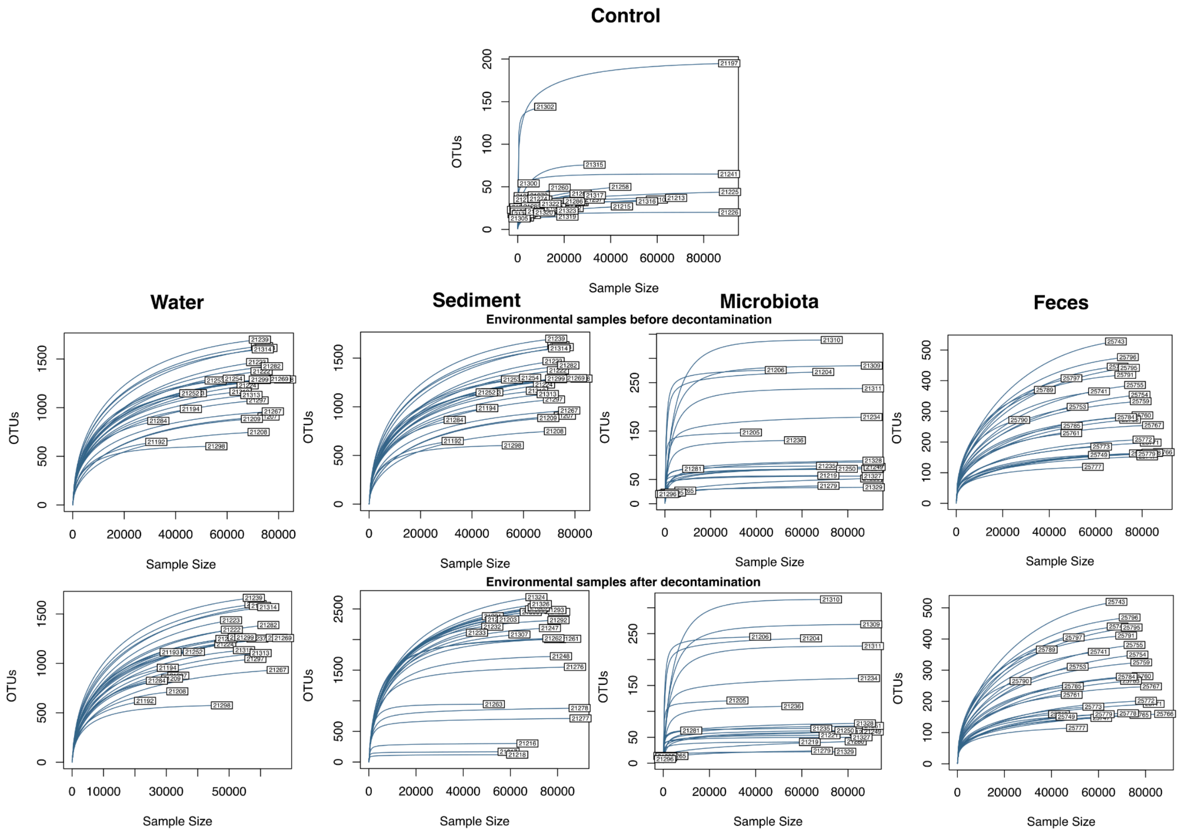

Figure S6. Rarefaction curves for control samples (upper row), environmental samples before (middle row), and after decontamination (bottom row).

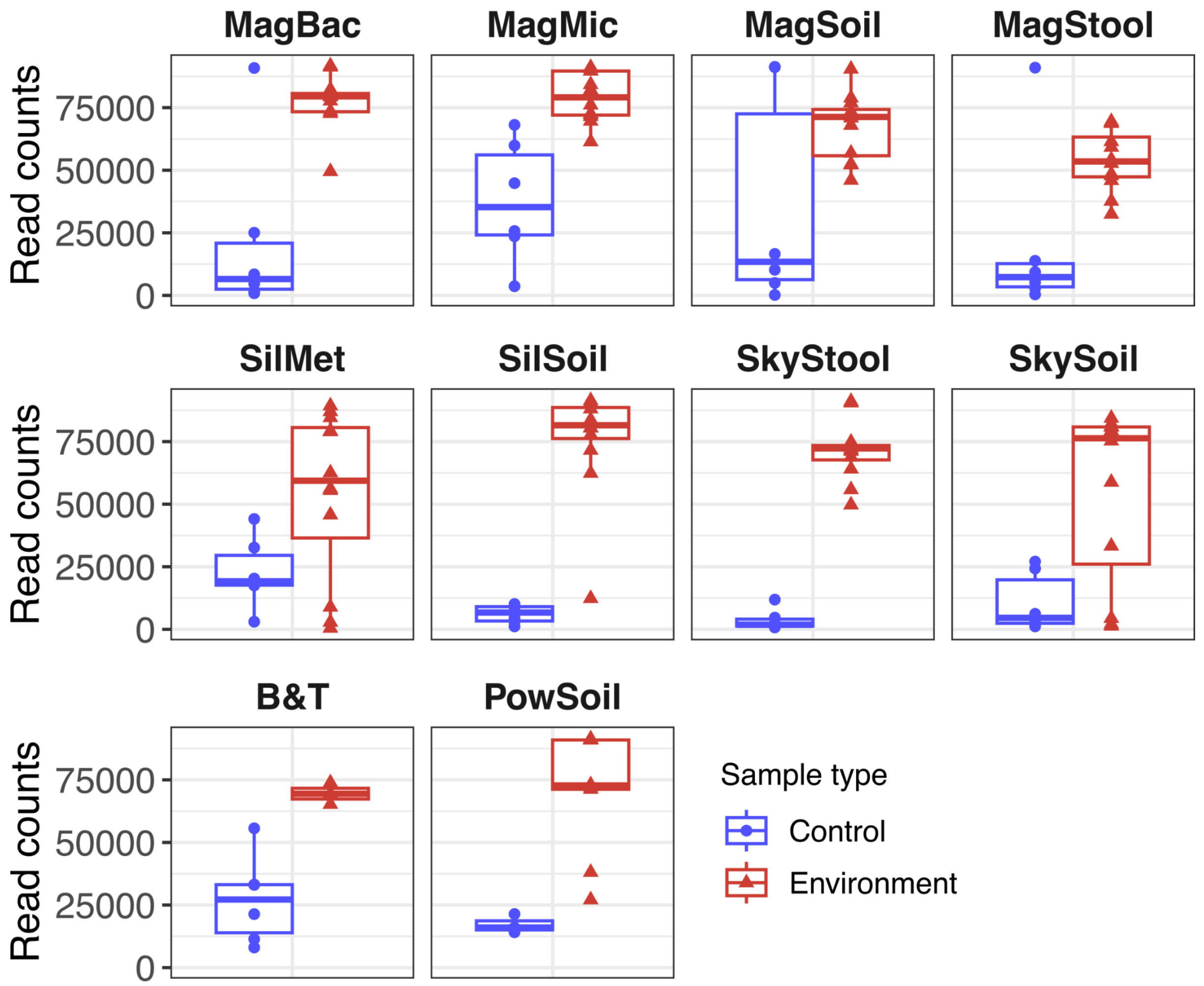

Figure S7. Total read counts after data processing with DADA2 (including denoising, merging, and chimera removal steps) for different DNA extraction kits. Blue and red box plots represent control ("kitome" and "splashome") and environmental samples, respectively. Individual samples are shown, respectively, with red dots and blue triangles. Data for three technical replicates are shown.

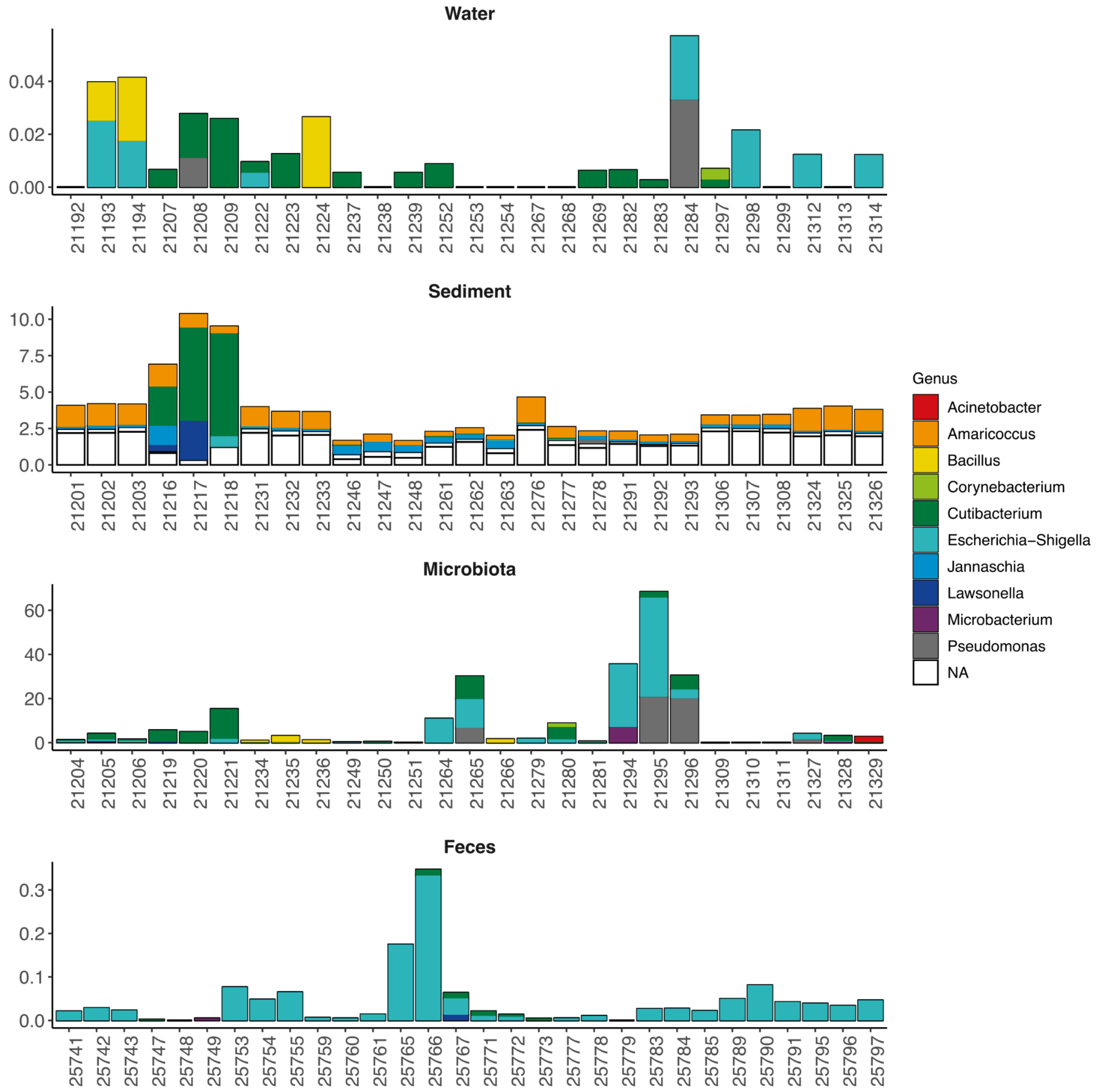

Figure S8. Contamination levels of environmental samples. Data is shown for all technical replicates independently. Genera with relative abundances >1% are shown.

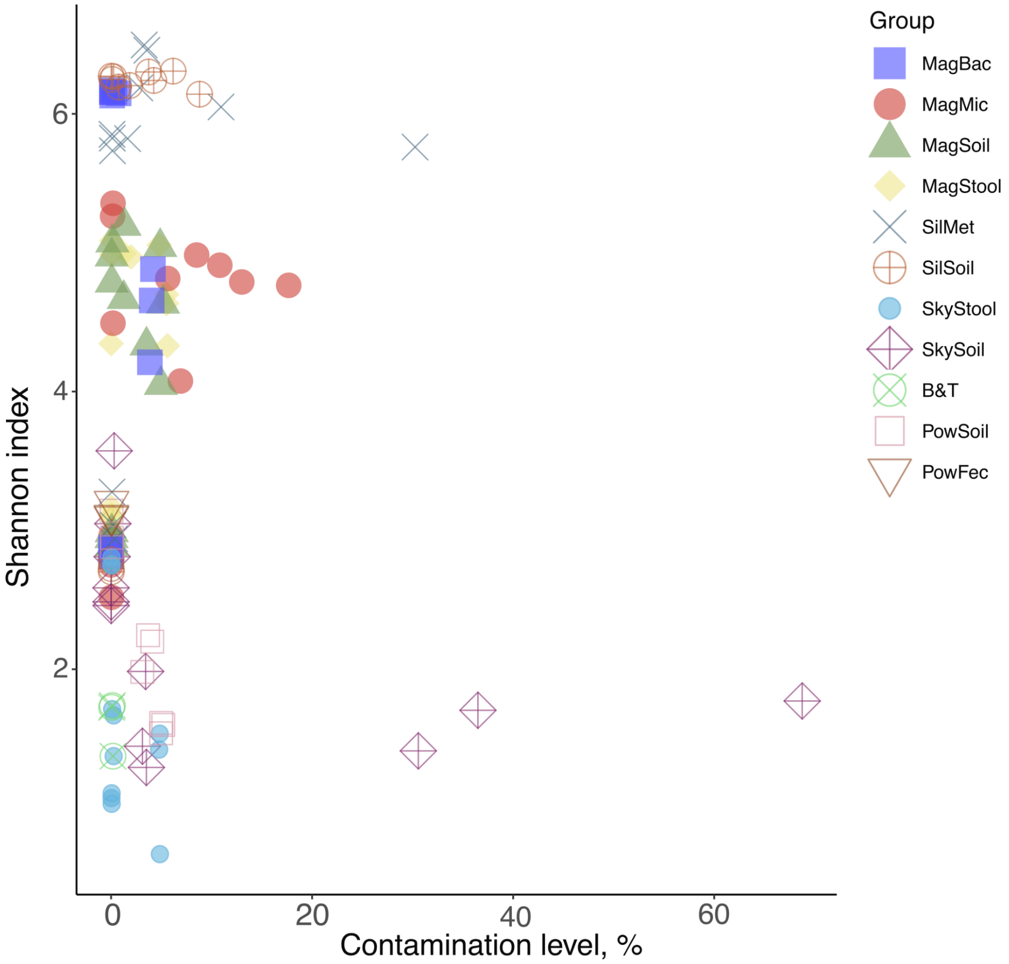

Figure S9. Contamination level and alpha diversity (Shannon index) of samples processed with different DNA extraction kits.

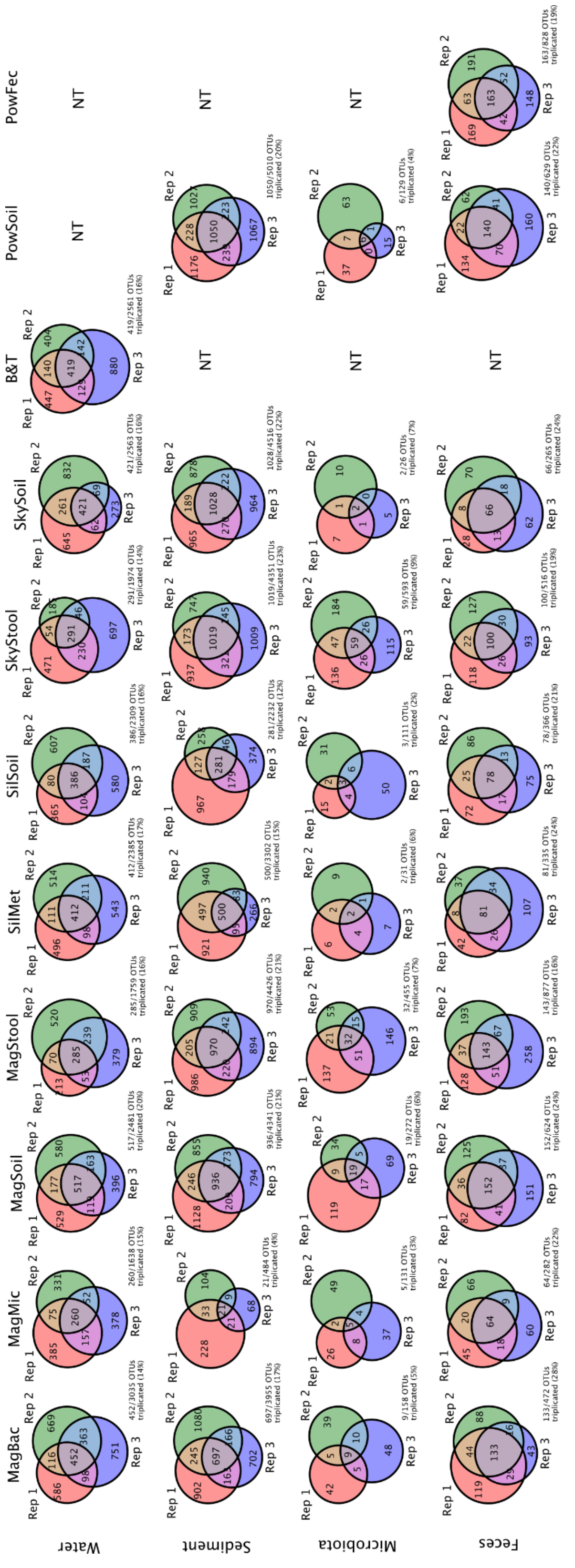

Figure S10. Reproducibility of DNA extraction kits. Venn diagrams representing the intersections of lists of non-zero abundant OTUs (OTUs with a non-zero abundance) for three technical replicates obtained with specified DNA-extraction kits. Below each diagram, reproducibility level is shown (%) as a fraction of non-zero OTUs found in all three replicates from the total number of unique non-zero OTUs found in at least one replicate. NT — not tested.

Supplementary Tables

Table S1. Quality and quantity of DNA purified from four types of samples by various DNA extraction kits. N/A — No results were obtained due to low DNA quality or the presence of PCR inhibitors; N/T — the combination of sample and kit was not tested.

Sample type		Kit										
		MagStool	MagMic	MagSoil	MagBac	SilMet	SilSoil	Sky-Soil	Sky-Stool	B&T	Pow-Soil	PowFec
Water	DNA amount, ng	5300± 1646	548,8± 10,4	1904± 498	2212± 270	1352± 462	315,6± 48,4	680,6± 107,6	1742± 1455	1178± 176	N/T	N/T
	Quality (260/280)	1,74± 0,11	1,21± 0,14	1,78± 0,09	1,69± 0,09	1,77± 0,22	1,94± 0,24	1,70± 0,11	1,69± 0,21	1,91± 0,35	N/T	N/T
	Quality (260/230)	1,48± 0,27	0,58± 0,28	1,65± 0,07	1,27± 0,44	1,68± 0,12	0,98± 0,38	N/A	1,23± 0,66	0,51± 0,11	N/T	N/T
	Fragmen-tation (DIN)	1,48± 0,27	0,72± 0,21	1,65± 0,07	1,27± 0,44	1,68± 0,12	1,02± 0,31	N/A	1,23± 0,66	0,51± 0,11	N/T	N/T
	Inhibition level	4,5± 0,2	7,31±0	N/A	N/A	N/A	3,48± 0,82	N/A	N/A	N/A	N/T	N/T
	Eukaryotic contami-nation	2,87± 0,52	0,81± 0,46	3,68± 1,13	N/A	1,48± 0,99	3,09± 0,69	2,85± 0,67	12,29± 9,62	13,77± 4,75	N/T	N/T
Sedi-ment	DNA amount, ng	2440± 440	30,68± 9,70	1446± 588	330,8± 64,4	45,16± 20,80	168,4± 80,8	368,8± 79,2	2040± 156	N/T	3380± 469	N/T
	Quality (260/280)	1,68± 0,04	1,25± 0,17	1,77± 0,05	1,38± 0,07	1,46± 0,05	1,73± 0,12	1,58± 0,61	1,35± 0,68	N/T	1,84± 0,04	N/T
	Quality (260/230)	1,53± 0,15	0,52± 1,86	1,53± 0,24	0,55± 0,03	0,73± 0,31	0,60± 0,22	0,96± 0,05	1,06± 0,34	N/T	0,47± 0,12	N/T
	Fragmen-tation (DIN)	6,5± 0,1	1±0	6,8± 0,2	1	1,43± 0,75	1,77± 0,35	6,0± 0,1	6,47± 0,12	N/T	6,07± 0,06	N/T
	Inhibition level	5,04± 0	N/A	N/A	N/A	N/A	11,18± 3,19	N/A	N/A	N/T	8,00± 1,30	N/T
	Eukaryotic contami-nation	6,61± 0,45	2,57± 0,76	6,40± 0,47	6,08± 0,18	5,45	6,83± 0,77	5,95± 2,11	N/A	N/T	5,78± 0,56	N/T
Micro-biota	DNA amount, ng	35± 11	19,88± 3,52	106,5±7 7,32	486,4± 101,5	432,8± 550,1	198,8± 79,2	36,32± 6,19	2256± 606	N/T	119,6± 56,9	N/T
	Quality (260/280)	1,39± 0,19	1,83± 1,14	1,45± 0,43	1,98± 0,05	1,59± 0.23	1,86± 0,14	1,63± 0,11	1,91± 0,05	N/T	1,92± 0,15	N/T
	Quality (260/230)	1,03± 0,26	0,86± 0,61	0,98± 0,24	1,68± 0,13	1,03± 0,33	1,08± 0,43	1,29	1,51± 0,11	N/T	0,04± 0,03	N/T
	Fragmen-tation (DIN)	1±0	1,3± 0,44	1±0	1±0	N/A	1±0	1±0	5,8	N/T	1±0	N/T
	Inhibition level	3,47± 2,59	N/A	N/A	N/A	0,58± 1,88	5,77± 0,119	1,83± 2,00	N/A	N/T	-0,13± 2,58	N/T
	Eukaryotic	1,39±	-0,41±	0,51±0,	4,46±	-1,25±	0,57±	-0,02±	7,61±	N/T	-1,72±	N/T

	contami-nation	0,52	1,29	64	0,49	1,23	1,40	0,84	5,82		1,09	
Feces	DNA amount, ng	1884± 462	1620± 697	1274± 393	3132± 475	632± 418	2304± 534	322± 75	10100± 4991	N/T	7900± 1179	13700± 2688
	Quality (260/280)	1,99± 0,12	2,09± 0,05	1,51± 0,09	1,63± 0,19	1,14± 0,04	1,91± 0,09	1,70± 0,03	1,88± 0,01	N/T	1,93± 0,03	1,92± 0.01
	Quality (260/230)	1,86± 0,13	1,53± 0,09	0,70± 0,05	0,86± 0,03	0,91± 0,64	1,46± 0,13	N/A	1,48± 0,59	N/T	1,66± 0,17	0,75± 0,10
	Fragmen-tation (DIN)	6,73± 0,15	1,17± 0,29	5,97± 0,06	1,43± 0,15	2,03± 1,00	1,17± 0,15	5,47± 0,31	5,87± 0,21	N/T	5,73± 0,06	5,50± 0,87
	Inhibition level	9,88± 1.96	15,76± 1,49	18,40±1 ,73	15,90± 1,36	N/A	16,52± 3,52	5,86± 5,79	N/A	N/T	8,62± 0,22	11,37± 0,94
	Eukaryotic contami-nation	5,87± 0,24	11,01± 0,43	10,74±0 ,47	15,68± 1,42	8,29	11,23± 1,72	8,63± 0,46	13,77	N/T	4,51± 0,65	6,58± 0.17

Table S2. Read count statistics for control and environmental samples. E/C — Environment / Control ratio.

Kit	Sample type	Mean read count	Median read count	Standard deviation	E/C median ratio	E/C mean ratio
B&T	Control	27114.5	27200	17488.111	2.552757	2.563487
B&T	Environment	69507.667	69435	4307.46		
MagBac	Control	21924	6540	34902.261	12.158257	3.536395
MagBac	Environment	77531.917	79515	10782.673		
MagMic	Control	37637.833	35272	24369.124	2.24277	2.105147
MagMic	Environment	79233.167	79107	9981.823		
MagSoil	Control	35739.833	13422	43322.321	5.311764	1.890363
MagSoil	Environment	67561.25	71294.5	13042.812		
MagStool	Control	20431.667	7288.5	34885.451	7.33937	2.640668
MagStool	Environment	53953.25	53493	12199.543		
PowSoil	Control	17176.667	16017	3783.236	4.542798	4.06197
PowSoil	Environment	69771.111	72762	22984.849		
SilMet	Control	22619	19185.5	14124.891	3.094472	2.39987
SilMet	Environment	54282.667	59369	33290.335		
SilSoil	Control	6142.667	6734.5	3738.924	12.106021	12.363767
SilSoil	Environment	75946.5	81528	21733.376		
SkySoil	Control	10669	4599	11793.132	16.59676	5.156622
SkySoil	Environment	55016	76328.5	34602.685		
SkyStool	Control	3718.167	1855	4231.785	38.966038	19.197857
SkyStool	Environment	71380.833	72282	11839.684		
